# Efficient and sustainable fixation of CO_2_ into 2-oxazolidinones utilizing ionic liquid functionalized SiO_2_ nanocomposites

**DOI:** 10.1186/s13065-025-01627-7

**Published:** 2025-09-30

**Authors:** Yulin Hu, Shiyao Lin, Xiaobing Liu

**Affiliations:** 1 College of Chemistry and Chemical Engineering, Anshun University, Anshun , 561000 China; 2 College of Chemistry and Chemical Engineering, Jinggangshan University, Ji’an, 343009 China

**Keywords:** Ionic liquids, CO_2_ cyclization reaction, 2-Oxazolidinones, Heterogeneous catalysis, High efficiency

## Abstract

Chemical fixation of CO_2_ into 2-oxazolidinones is of significance because of the important application in the chemical synthesis. In this study, a series of ionic liquid functionalized SiO_2_ nanocomposites were synthesized and developed as heterogeneous catalysts for the CO_2_ cyclization reaction. The resulting SiO_2_-MILZrCl_5_ behaved as high-efficiency heterogeneous catalyst for solvent-additive-free CO_2_ cyclization at 70 °C and 0.1 MPa CO_2_. A high yield of 93% with selectivity of 99.4% over 5-methyl-3-phenyloxazolidin-2-one was attained. Furthermore, a wide range of epoxides were transformed with high yields and excellent selectivities under atmospheric pressure. Additionally, SiO_2_-MILZrCl_5_ could serve as a durable and recoverable heterogeneous catalyst for the reaction, which could be used for five cycles without significant loss of catalytic activity. This study offers novel insights for the development of green catalysts for the efficient CO_2_ fixation.

**Graphical Abstract:**

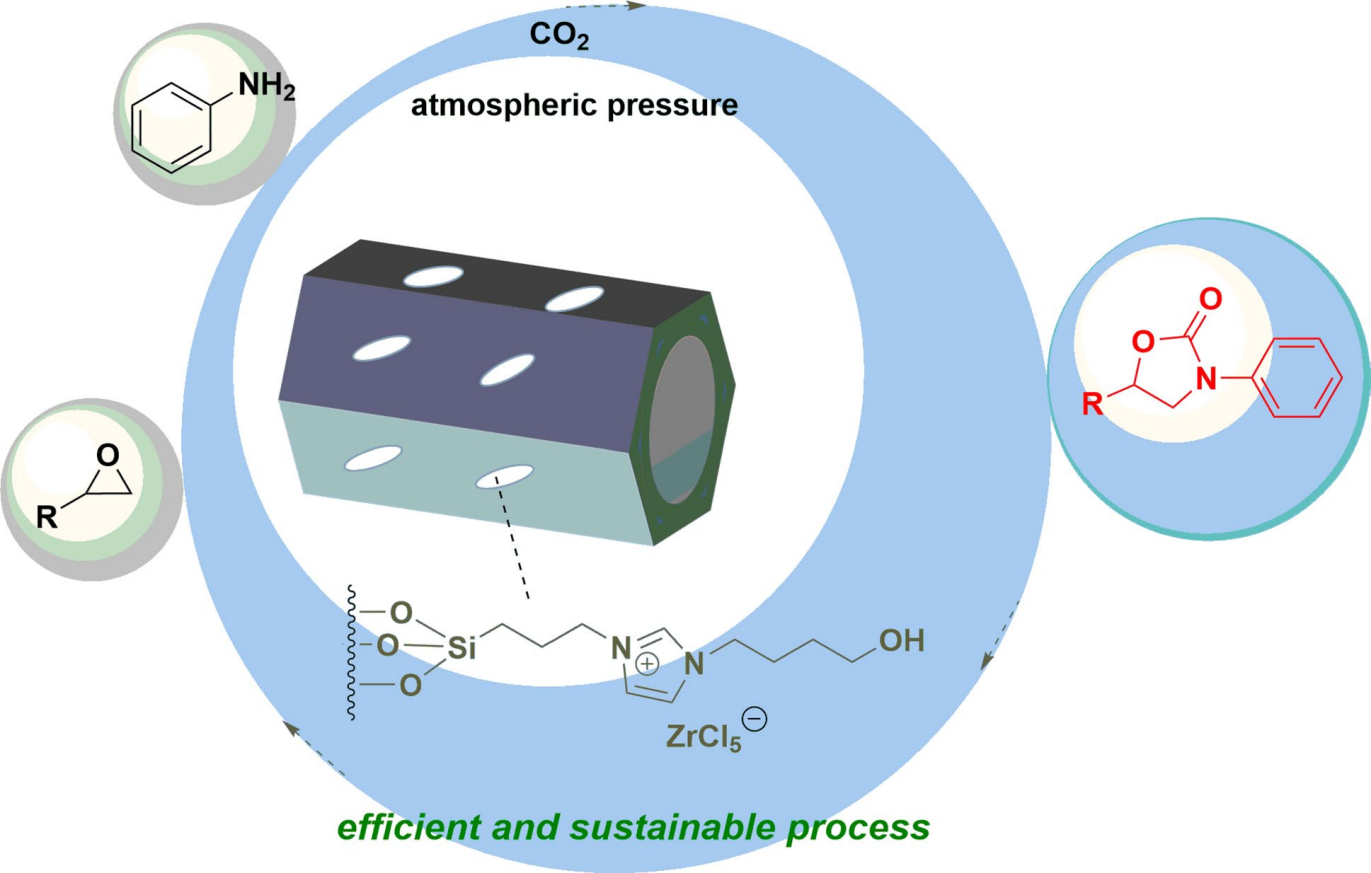

**Supplementary Information:**

The online version contains supplementary material available at 10.1186/s13065-025-01627-7.

## Introduction

Carbon dioxide (CO_2_) emissions have emerged as one of the most pressing environmental challenges, and various strategies are being explored to regulate ambient CO_2_ levels and advance carbon peaking/neutrality objectives [1–3]. The utilization of CO_2_ as a sustainable C1 feedstock for converting into value-added chemicals not only consumes the emitted CO_2_ but also reduces the dependence on the fossil fuels [4–8]. Particular attention has focused on the synthesis of five-membered 2-oxazolidinones by the exploration of aziridines, isocyanates, amino alcohols, aniline and epoxides via cycloaddition reactions with CO_2_ [9–14]. Considering the starting material availability and economic viability, the most compatible approach is the three-component reaction of CO_2_, epoxides and amines [11]. To date, numerous catalytic systems have been developed for the CO_2_ conversion including 1,1,3,3,-tetramethylguanidine/visible light irradiation [15], Br^−^Ph_3_^+^P-PEG_600_-P^+^Ph_3_Br^−^ [16], [Tb_8_(acac)_6_(L)_2_(µ_3_-O)_6_(µ_2_C_2_H_5_O)_4_(µ_2_-Hacac)_2_] [17], Ce@PCN-777 [18], Mg-porphyrin [19], PVA-DFNT/Ni [20], K_3_PO_4_ [21], UiO-66-40 [22], and others [23–26]. Although significant progress has been made, most reported catalyst systems usually require harsh reaction conditions (elevated temperatures and pressures), use of expensive reagents and co-catalysts to overcome the inherent thermodynamic stability and kinetic inertness of CO_2_ molecules. Consequently, there is an urgent need to develop more efficient catalysts with task-specific catalytic active sites for the chemical fixation of CO₂ into 2-oxazolidinones under mild conditions.

**Scheme 1:**
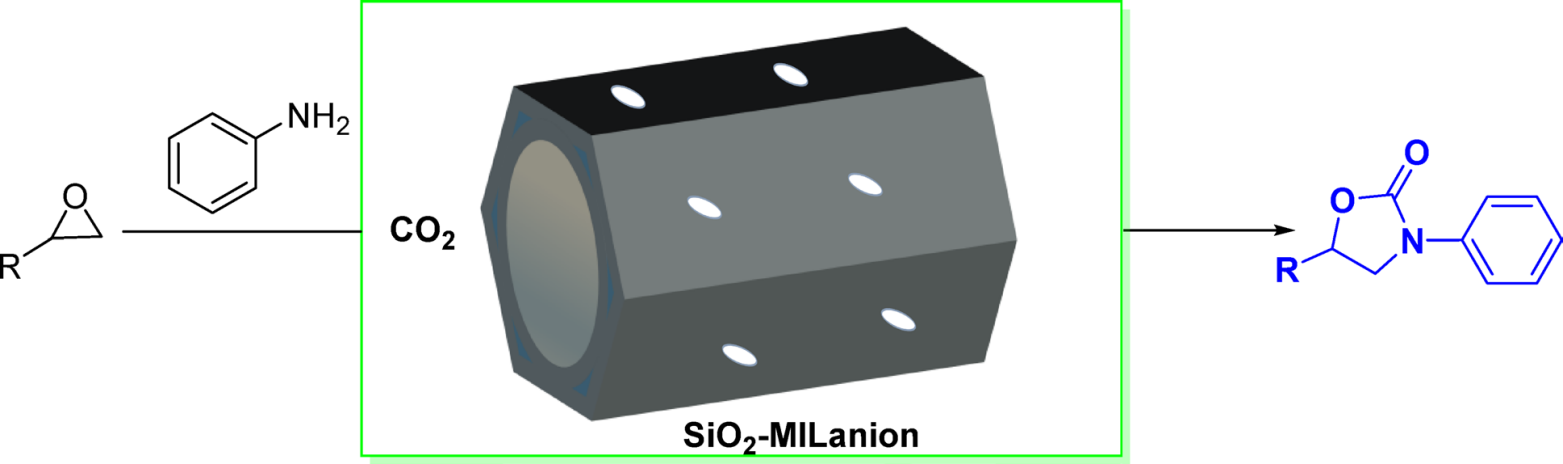
Catalytic chemical fixation of carbon dioxide into 2-oxazolidinones

Ionic liquids are appealing chemical complexes that are formed from ions, which are widely used in various fields and sciences due to different properties such as non-evaporation, non-burnable, stability, etc [27–34]. Ionic liquids can be designed by more than one type of cation and anion and can act as efficient catalysts for the transformation of CO_2_ to 2-oxazolidinones [35–38]. Nevertheless, homogeneous characteristics of ILs complicate product separation and catalyst recovery, requiring the development of heterogeneous IL-based catalytic systems. Immobilization of ILs on various porous solid supports is a general strategy to construct ILs-based heterogeneous catalyst. Silica, particularly porous SiO_2_ is an important class of porous materials that have shown promising catalytic activity [39, 40]. Consequently, modifying of SiO_2_ by using homogeneous ILs, would be desirable for different purposes including heterogeneous catalysis, which possessed their special features of sufficient contact and easy separation from the reaction mixture in the chemical reactions [41–45]. Among the various ionic liquids, metal-containing imidazolium ILs, have revealed significant potential for catalysis applications, which showcase some promising features in CO_2_ capture and transformation [46–50]. In continuation of research on IL-functionalized silica-based catalytic systems and CO_2_ fixation reactions, in this study, we propose a three-step synthetic strategy for construction of silica gel supported imidazolium-based ionic liquids with functional anion active sites and cation groups. The as-prepared supported nanocomposites exhibit a considerable surface area, well-distributed ionic sites and abundantly functional groups. The structural advantages significantly enhance CO_2_ adsorption and activation, thereby promoting the coupling of CO_2_, epoxides and amines to efficient construction of 2-oxazolidiones (Scheme 1) under mild conditions with good catalytic efficiency and recyclability.

## Experimental

### Preparation of SiO_2_-MILanion (Scheme 2)

Typically, 4-(1 H-imidazol-1-yl)butan-1-ol (0.15 mol) and (3-chloropropyl) triethoxysilane (0.15 mol) were dissolved in solvent of toluene (120 mL). The reaction was conducted at 105 °C with condensation reflux and magnetic stirring for 24 h. After the reaction, the solvent was removed by liquid-liquid separation, followed by vacuum drying at 70 °C for 5 h to afford MILCl **I**. Subsequently, SmCl_3_ or ZrCl_4_ or CoCl_2_ or EuCl_3_ or FeCl_3_ (0.1 mol), **I** (0.1 mol) and CH_3_CN (80 mL) were stirred vigorously at 40 °C for 24 h. Then, the solvent was removed and the crude ionic liquid was washed with ether three times and dried under vacuum at 60 °C for 5 h to afford MILanion **II**. Finally, **II (**4.5 g**)** and 18 mL tetraethylorthosilicate (TEOS) were dissolved in solvent of EtOH (20 mL), and the mixture was stirred at 60 °C for 4 h. Then concentrated hydrochloric acid (7.8 mL) and deionized water (7.2 mL) were gradually added to the mixture, after which it was aging at a temperature of 60 °C for 12 h. The resulting of solid material was dried under vacuum at 120 °C for 6 h to give the supported ILs SiO_2_-MILanion.

**Scheme 2:**
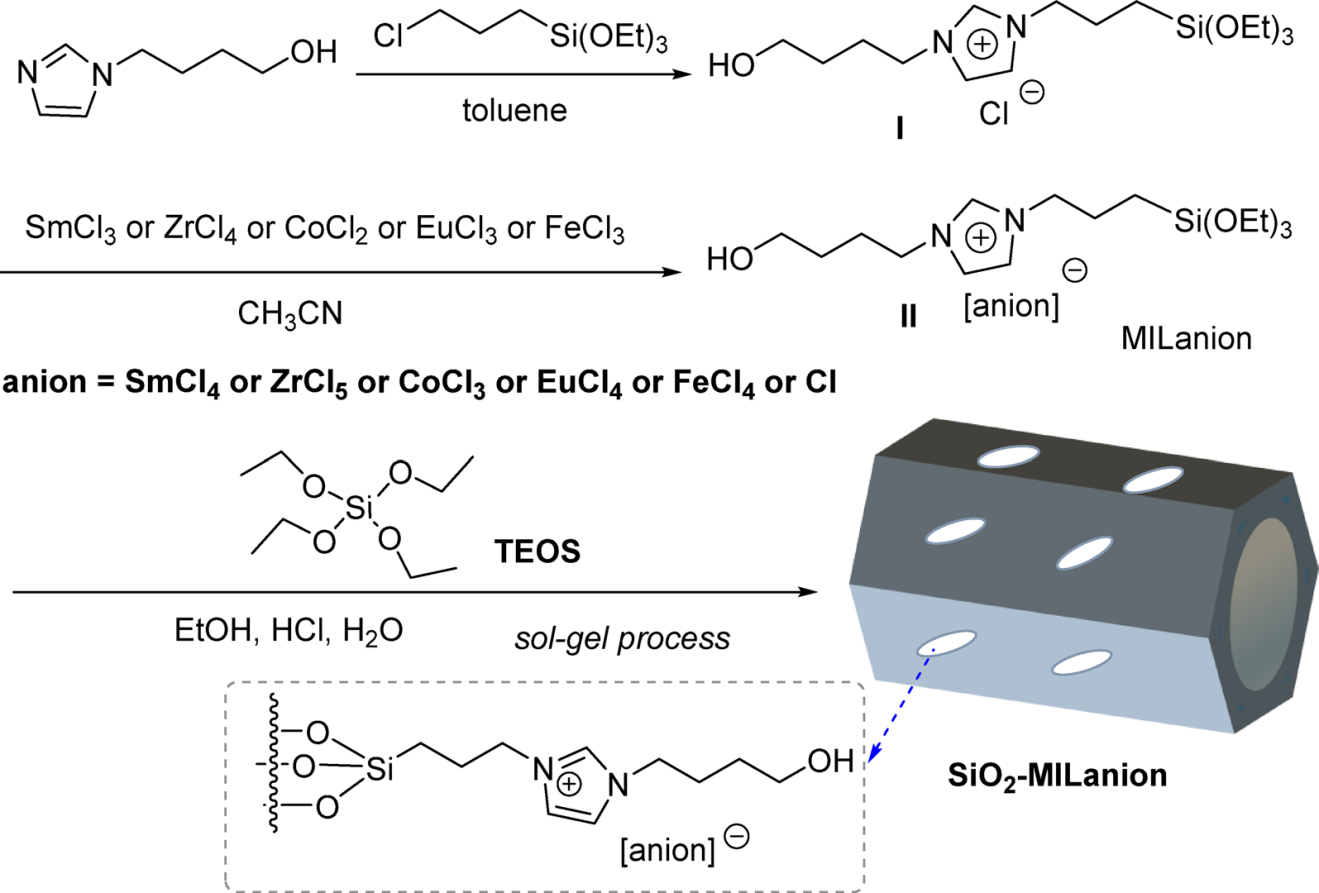
Preparation of SiO_2_-MILanion

### General procedure for fixation of CO_2_ into 2-oxazolidinones

In a standard procedure, epoxide (10 mmol), aniline (4.5 mmol) and SiO_2_-MILZrCl_5_ (0.15 g) were added to a 50 mL stainless-steel autoclave. CO_2_ was slowly blown into the autoclave to remove the preexisted air, and the pressure of carbon dioxide was maintained at 0.1 MPa (CO_2_ balloon). Then, the reaction mixture was magnetically stirred at 70 °C for 3 h. The progress of the reaction was monitored by gas chromatograph (GC, Agilent 7890 A) with a flame ionization detector (FID) to quantify the conversion and selectivity. After completion of the reaction, 15 ml ethanol was poured and the solid catalyst was removed from the reaction mixture by simple filtration and then rinsed multiple times with ethanol, dried under vacuum at 60 °C, and reused in the next cycle. The filtrate was concentrated by rotary evaporation and the crude product was purified by column chromatography over silica gel (petroleum ether/ethyl acetate = 5/1). The analytical data obtained were rigorously compared with already published values [16–20] to confirm the identification of the compounds.

### Spectral data of 2-oxazolidinones

5-methyl-3-phenyl oxazolidin-2-one (**a**) [13]: ^1^H NMR (400 MHz, CDCl_3_) (δ/ppm): 2.92 (d, 3 H, CH_3_), 3.47 (t, 1H, CH_2_), 3.93 (t, 1H, CH_2_), 5.46 (m, 1H, CH), 7.43–7.29 (m, Ar-H, 5 H); ^13^C NMR (400 MHz, CDCl_3_) (δ/ppm): 21.1, 52.3, 69.8, 118.2, 124.3, 129.4, 138.5, 155.2; Elemental analysis for C_10_H_11_NO_2_: C, 67.73; H, 6.20; N, 7.84; O, 18.01. Found C, 67.78; H, 6.26; N, 7.90; O, 18.06.

3-phenyloxazolidin-2-one (**b**) [14]: ^1^H NMR (400 MHz, CDCl_3_) (δ/ppm): 4.06 (t, CH_2_, 2 H), 4.51 (t, CH_2_, 2 H), 7.16 (m, Ar-H, 1H), 7.37–7.52 (m, Ar-H, 4 H); ^13^C NMR (400 MHz, CDCl_3_) (δ/ppm): 45.4, 61.6, 118.3, 124.4, 129.3, 138.5, 155.3; Elemental analysis for C_9_H_9_NO_2_: C, 66.23; H, 5.51; N, 8.54; O, 19.55. Found: C, 66.25; H, 5.56; N, 8.58; O, 19.61.

5-butyl-3-phenyl oxazolidin-2-one (**c**) [14]: ^1^H NMR (400 MHz, CDCl_3_) (δ/ppm): 0.95 (t, CH_3_, 3 H), 1.28–1.42 (m, 2 H, CH_2_), 1.53–1.57 (m, CH_2_, 2 H), 3.36–3.24 (m, CH_2_, 2 H), 3.43 (t, CH_2_, 1H), 3.94 (t, 1H, CH_2_), 5.46 (m, 1H, CH), 7.41–7.28 (m, Ar-H, 5 H); ^13^C NMR (400 MHz, CDCl_3_) (δ/ppm): 14.2, 22.6, 26.8, 34.9, 50.8, 73.4, 118.4, 124.2, 129.3, 138.7, 155.2; Elemental analysis for C_13_H_17_NO_2_: C, 71.15; H, 7.78; N, 6.32; O, 14.54. Found C, 71.21; H, 7.81; N, 6.39; O, 14.59.

5,5-dimethyl-3-phenyl oxazolidin-2-one (**d**) [15]: ^1^H NMR (400 MHz, CDCl_3_) (δ/ppm): 1.59 (s, CH_3_, 6 H), 3.78 (s, CH_2_, 2 H), 7.19 (m, Ar-H, 1H), 7.39–7.54 (m, Ar-H, 4 H); ^13^C NMR (400 MHz, CDCl_3_) (δ/ppm): 22.3, 53.4, 71.7, 118.6, 124.5, 129.1, 138.4, 155.3; Elemental analysis for C_11_H_13_NO_2_: C, 69.07; H, 6.82; N, 7.24; O, 16.71. Found C, 69.09; H, 6.85; N, 7.32; O, 16.73.

3,5-diphenyloxazolidin-2-one (**e**) [15]: ^1^H NMR (400 MHz, CDCl_3_) (δ/ppm): δ = 3.95 (t, CH_2_, 2 H), 4.37 (m, CH_2_, 2 H), 5.65 (t, CH, 1H), 7.15 (m, Ar-H, 1H), 7.37–7.39 (m, Ar-H, 3 H), 7.43–7.46 (m, Ar-H, 4 H), 7.55–7.58 (m, Ar-H, 2 H); ^13^C NMR (400 MHz, CDCl_3_) (δ/ppm): 52.8, 74.3, 118.4, 124.4, 125.9, 129.1, 129.3, 138.2, 138.3, 154.9; Elemental analysis for C_15_H_13_NO_2_: C, 75.24; H, 5.43; N, 5.82; O, 13.35. Found C, 75.30; H, 5.48; N, 5.85; O, 13.37.

## Results and discussion

**Fig. 1:**
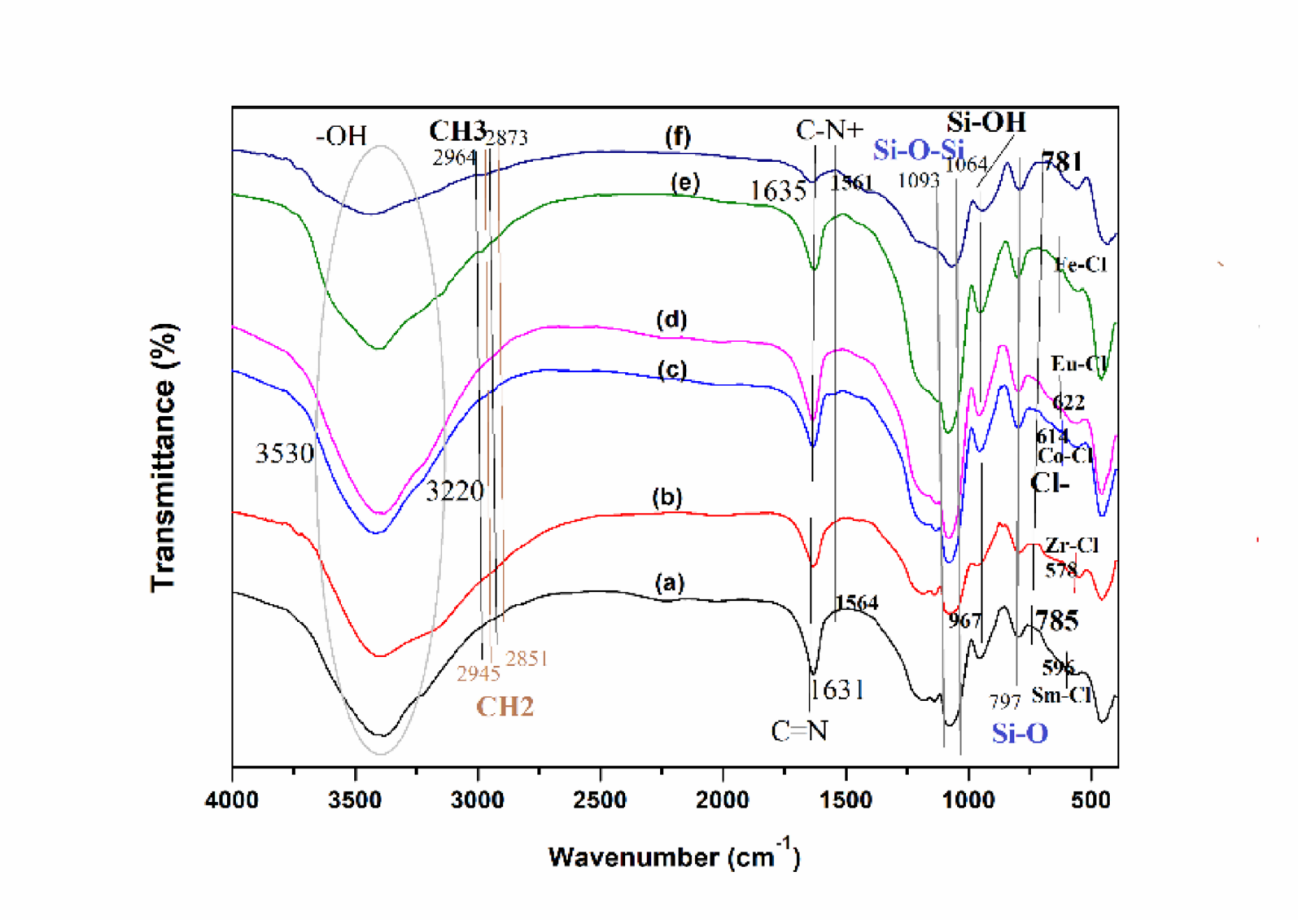
FT-IR spectra of (a) SiO_2_-MILSmCl_4,_ (b) SiO_2_-MILZrCl_5_, (c) SiO_2_-MILCoCl_3_, (d) SiO_2_-MILEuCl_4_, (e) SiO_2_-MILFeCl_4_, and (f) SiO_2_-MILCl

The chemical structure and functionality of the as-synthesized ionic liquid functionalized SiO_2_ nanocomposites are investigated via FT-IR spectroscopy (Fig. 1). The bands near 3530 –3220 cm^− 1^, and 967 cm^− 1^ are attributable to O-H stretching vibrations and bending vibrations of Si-OH groups, respectively. The bands at about 1093 –1064 cm^− 1^, and 797 cm^− 1^ attributed to the asymmetric and symmetric stretching vibrations of Si-O-Si groups, respectively [44–47]. The bands at around 2964 –2839 cm^− 1^ are attributed to the stretching vibration of CH_3_ and CH_2_ groups. All nanocomposites exhibit the featured bands at 1635 –1631 cm^− 1^ and 1564 − 1561 cm^− 1^ corresponding to the C = N and C-N^+^ stretching vibrations of the imidazole ring, which confirm the presence of imidazolinium-based ionic moieties within the framework [27–29]. The bands in the 781–785 cm^− 1^ region are assigned to the stretching vibrations of the Cl^−^ anion. The bands around 596 cm^− 1^, 578 cm^− 1^, 614 cm^− 1^, 622 cm^− 1^, 629 cm^− 1^ attributed to the stretching vibrations of Sm-Cl, Zr-Cl, Co-Cl, Eu-Cl, Fe-Cl, respectively [51–55]. Moreover, the FT-IR spectra of MILSmCl_4_, MILZrCl_5_, MILCoCl_3_, MILEuCl_4_, MILFeCl_4_, MILCl and SiO_2_ exhibit characteristic absorption bands corresponding to specific functional groups (Fig. S1), indicating that the chemical structure of their frameworks are virtually identical. The above results indicated the successful construction of these nanocomposites.

**Fig. 2:**
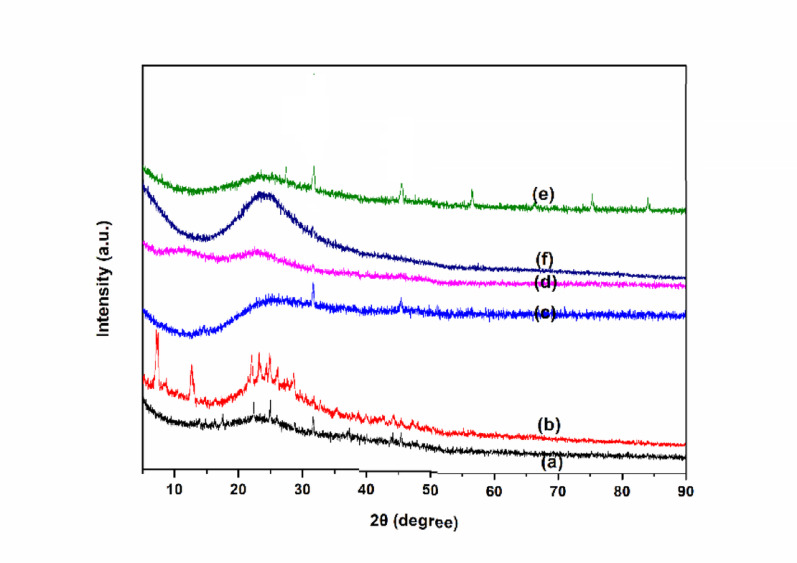
XRD pattern of (a) SiO_2_-MILSmCl_4,_ (b) SiO_2_-MILZrCl_5_, (c) SiO_2_-MILCoCl_3_, (d) SiO_2_-MILEuCl_4_, (e) SiO_2_-MILFeCl_4_, and (f) SiO_2_-MILCl

**Fig. 3:**
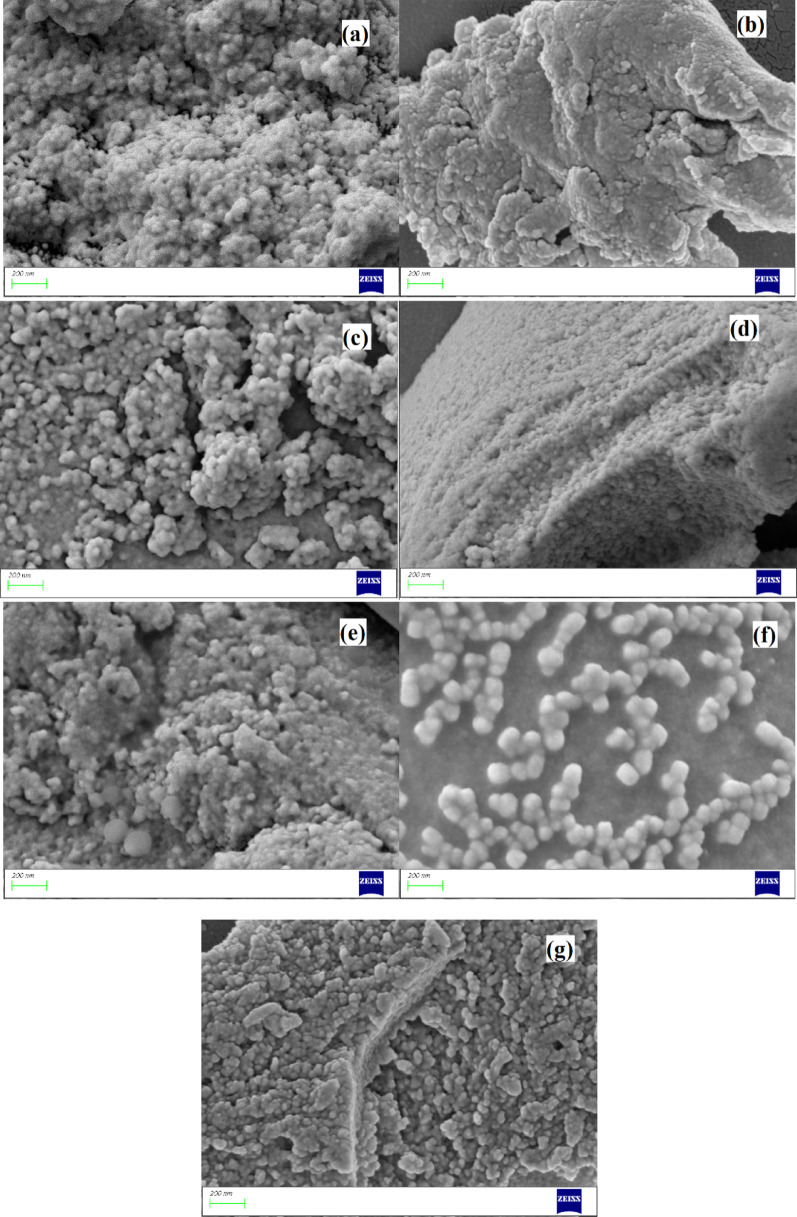
SEM images of (a) SiO_2_-MILSmCl_4,_ (b) SiO_2_-MILZrCl_5_, (c) SiO_2_-MILCoCl_3_, (d) SiO_2_-MILEuCl_4_, (e) SiO_2_-MILFeCl_4_, (f) SiO_2_-MILCl and (g) five times recovered SiO_2_-MILZrCl_5_

**Fig. 4:**
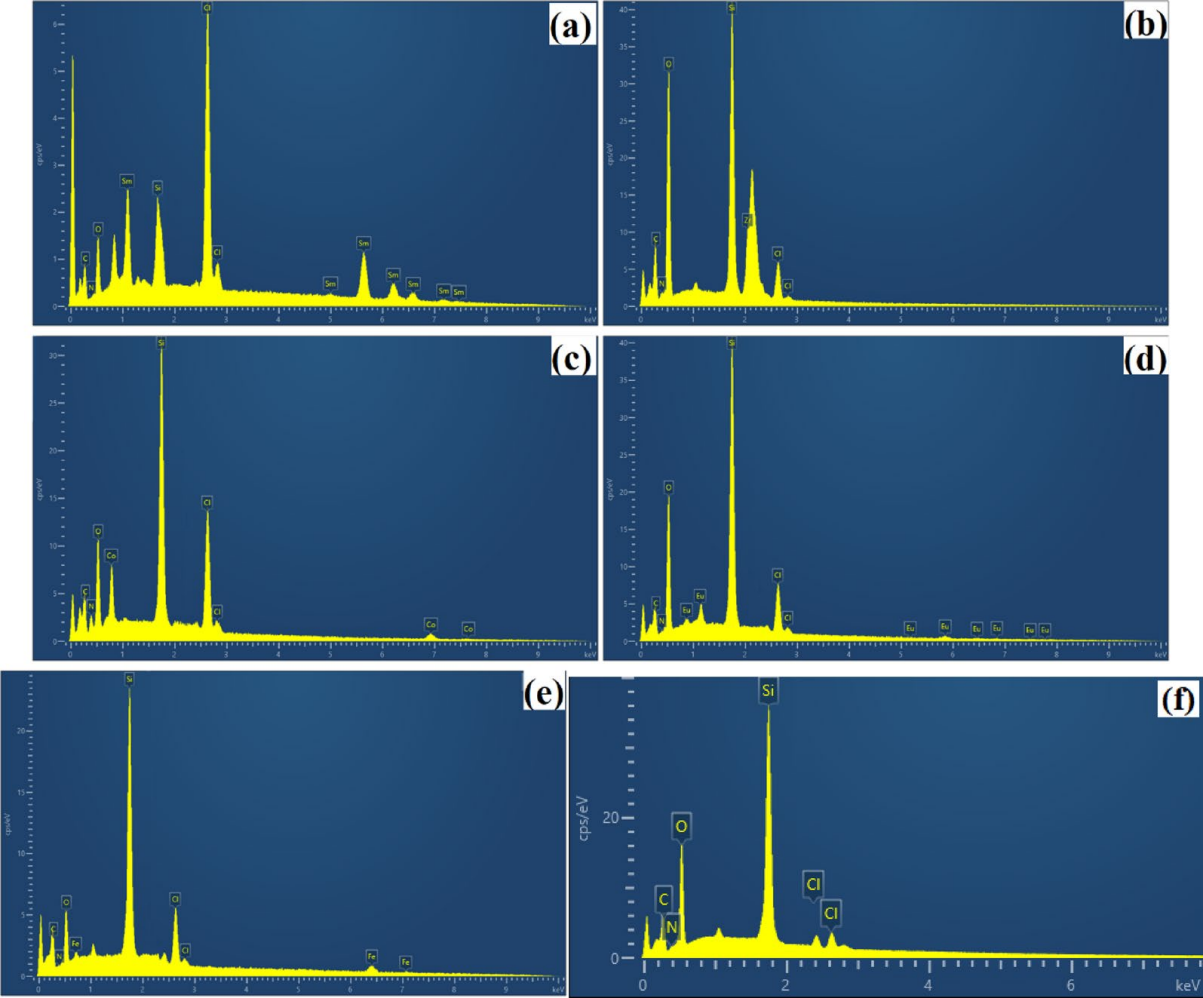
EDX images of (a) SiO_2_-MILSmCl_4,_ (b) SiO_2_-MILZrCl_5_, (c) SiO_2_-MILCoCl_3_, (d) SiO_2_-MILEuCl_4_, (e) SiO_2_-MILFeCl_4_, and (f) SiO_2_-MILCl

XRD spectra of the ionic liquid functionalized SiO_2_ nanocomposites are shown in Fig. 2. The diffraction peaks at 2*θ* = 21°-26° attributed well to the (022) plane of amorphous silica for all samples (JCPDS Card No. 29–0085). The XRD data, featuring a broad, featureless hump at 2θ = 4.3°, 8.3°, 11.0°, and 24.7°, attested to the characteristic diffraction peaks of Zr-Cl [56] (Fig. 2b). The diffraction peaks at 2*θ* = 26.7°, 30.3°, 45.2°, 56.7°, attested to the characteristic diffraction peaks of Fe-Cl [57] (Fig. 2e). Additionally, no obvious peaks compared to organic ionic liquid species were observed, indicating a reduction in crystallinity and well dispersion on the silica surface. SEM images are used to characterize the morphology of the ionic liquid functionalized SiO_2_ nanocomposites (Fig. 3). All the nanocomposites showed some aggregations on the surface of nanoparticles and displayed spherical morphology and typical uniformity, with dimensions of nanoparticles from several hundred nanometers to a few micrometers. The Energy-dispersive X-ray analysis (EDX) confirmed the elemental composition of the synthesized nanocomposites (Fig. 4 and Table S1). In addition, the elemental mapping of the nanocomposites exhibits a uniform dispersion of the corresponding elements (C, N, O, Si, Cl, Sm or Zr or Co or Eu or Fe) among the surfaces, indicating the elements are homogeneously distributed throughout the imidazolium-based porous framework (Fig. S2).

**Fig. 5:**
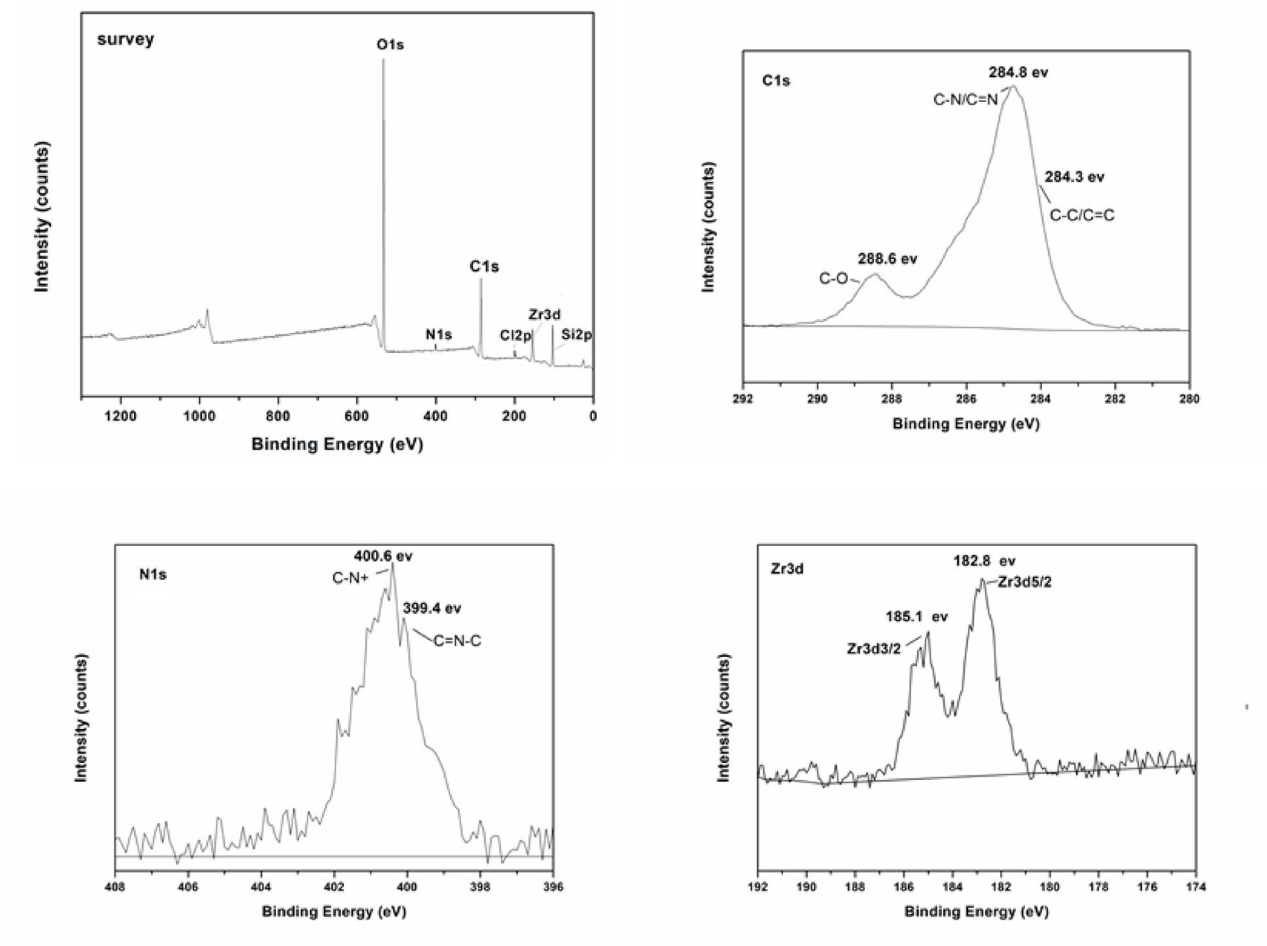
XPS spectra of survey, C1s, N1s, Zr3d of SiO_2_-MILZrCl_5_ catalyst

**Fig. 6:**
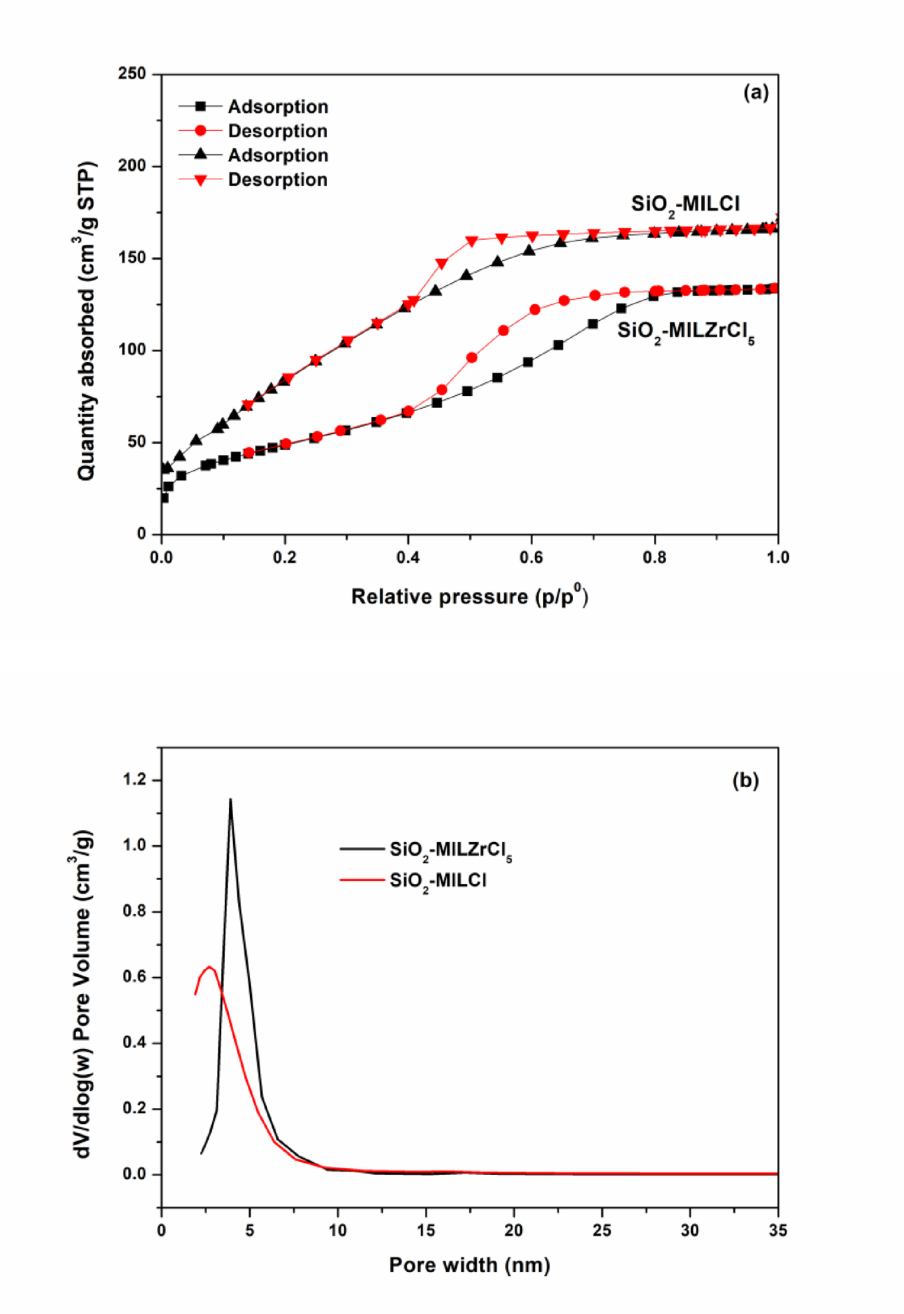
(a) N_2_ adsorption–desorption isotherms and (b) pore size distribution curves of SiO_2_-MILZrCl_5_ and SiO_2_-MILCl

**Fig. 7:**
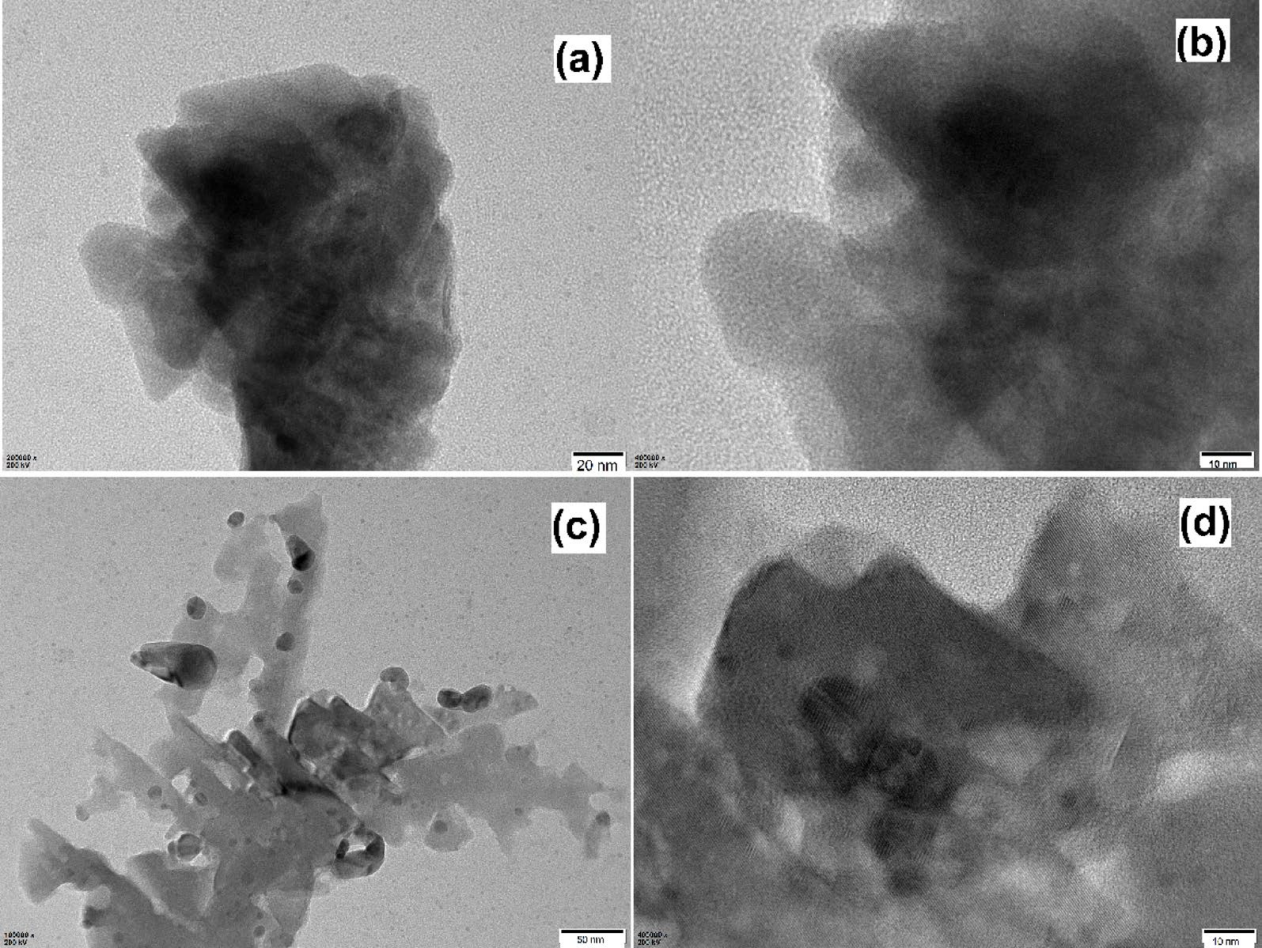
TEM images of (a, b) SiO_2_-MILZrCl_5_ and (c, d)SiO_2_-MILCl

XPS analysis is further utilized to characterize the surface composition of SiO_2_-MILZrCl_5_ catalyst. The full survey XPS spectra revealed the distinct peaks C1s, N1s, O1s, Cl2p, Zr3d, and Si2p peaks in SiO_2_-MILZrCl_5_ (Fig. 5 and Fig. S3), and the peaks appeared at 284.8 eV, 400.6 eV, 532.9 eV, 198.2 eV, 182.8 eV, and 103.5 eV, respectively [43–47]. In the N 1s XPS spectra of SiO_2_-MILZrCl_5_, the signals at 400.6 and 399.4 eV are attributed to the delocalized nitrogen cation and the non-ionic nitrogen of the imidazolium ring, respectively [58]. Similarly, in the C1s XPS spectra of SiO_2_-MILZrCl_5_, the signals at 288.6, 284.8 and 284.3 eV, are attributed to C-O, C − N/C = N, and C − C/C = C bonds, respectively [59]. The signals for Zr3d in SiO_2_-MILZrCl_5_ appeared at 185.1 eV (3d3/2) and 182.8 eV (3d5/2) [60], are assigned to free ZrCl_5_⁻ anions in SiO_2_-MILZrCl_5_. The XPS results further verified the supposed composition of the elements of the SiO_2_-MILZrCl_5_ catalyst. The porosity of SiO_2_-MILZrCl_5_ catalyst and non-metal containing ionic liquid functionalized SiO_2_ nanocomposite SiO_2_-MILCl are investigated through N_2_ adsorption-desorption isotherms (Fig. 6). Both SiO_2_-MILZrCl_5_ and SiO_2_-MILCl exhibit typical type IV isotherm with hysteresis loops at high relative pressure (p/p^0^ = 0.4–0.9), indicating the presence of porous structure. As displayed in Table S2, the specific surface area and pore volume of SiO_2_-MILZrCl_5_ shows significant decrease compared to SiO_2_-MILCl (182.54 m^2^ g^− 1^ and 0.26 cm^3^ g^− 1^ vs. 279.23 m^2^ g^− 1^ and 0.38 cm^3^ g^− 1^), whereas the average pore size shows no significant change (4.56 nm vs. 4.17 nm). This phenomenon may be attributed to the further Zirconium chloride anion ionic liquid functionalized in the silica framework. The predominant pore size of SiO_2_-MILZrCl_5_ is 4.56 nm, and the catalyst surface area presents a good surface area of 182.54 m^2^ g^− 1^, which could be useful for the forthcoming catalytic reactions. Moreover, TEM images of the representative samples SiO_2_-MILZrCl_5_ (Fig. 7a, b) and SiO_2_-MILCl (Fig. 7c, d) further confirmed the ionic liquid/silica nanocomposites structure, showing the uniform dispersion of ionic moieties in the framework. Additionally, the presence of numerous pores in the nanocomposites was observed, consistent with the analysis obtained from N_2_ adsorption isotherms.

**Fig. 8:**
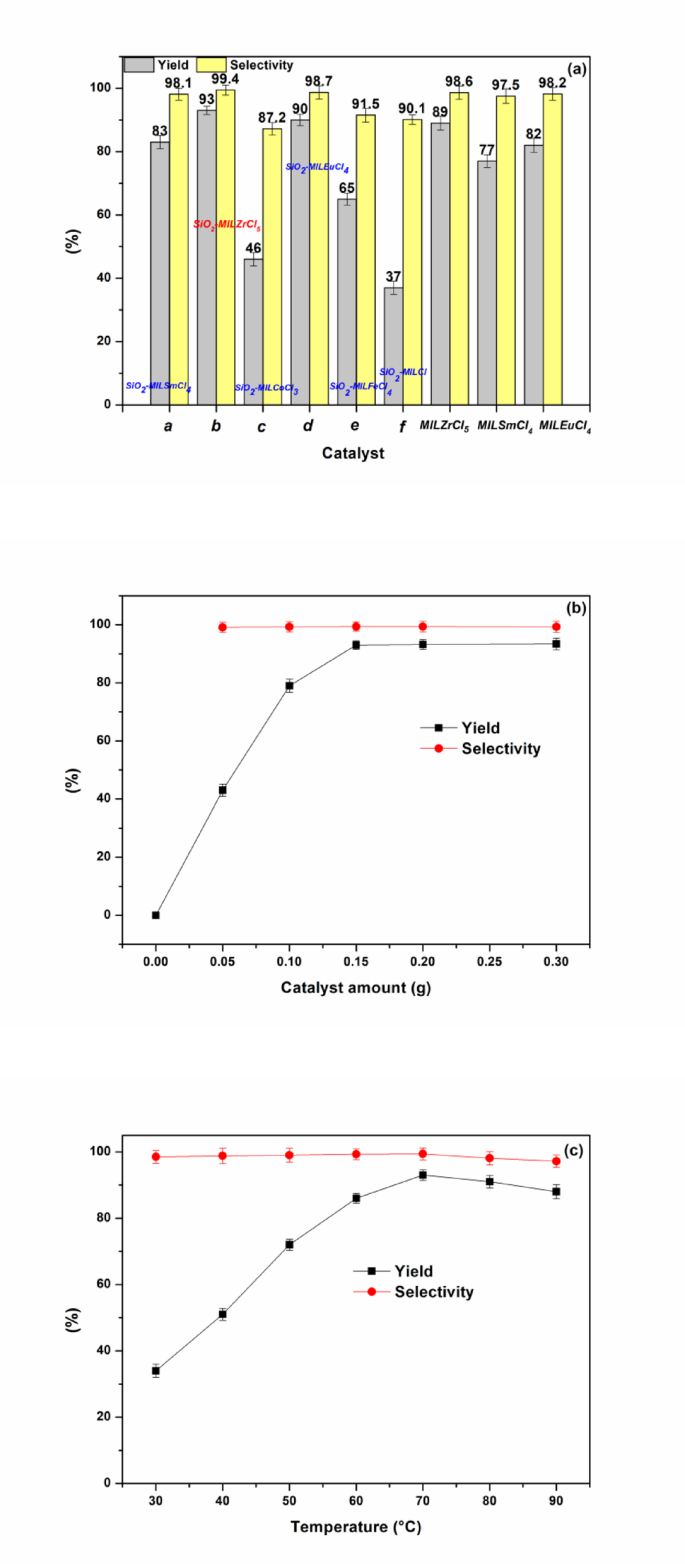

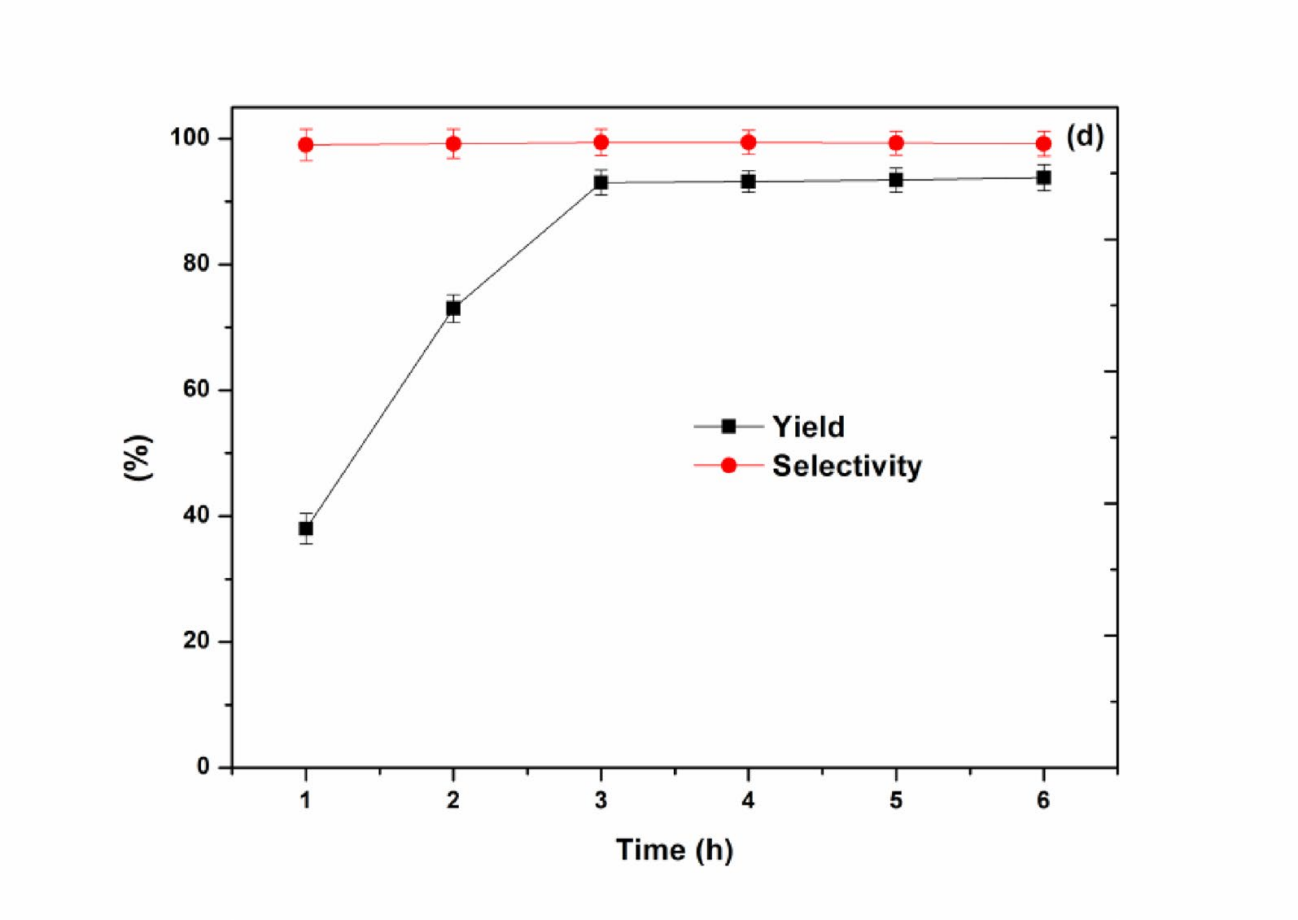
(a) Catalysts screening (10 mmol propylene oxide, 4.5 mmol aniline, 0.15 g catalyst, 0.1 MPa CO_2_, 70 °C, 3 h), (b) effect of catalyst amount (10 mmol propylene oxide, 4.5 mmol aniline, 0.1 MPa CO_2_, 70 °C, 3 h), (c) effect of temperature (10 mmol propylene oxide, 4.5 mmol aniline, 0.15 g SiO_2_-MILZrCl_5_, 0.1 MPa CO_2_, 3 h), (d) effect of reaction time on the reaction (10 mmol propylene oxide, 4.5 mmol aniline, 0.15 g SiO_2_-MILZrCl_5_, 0.1 MPa CO_2_, 70 °C)

The as prepared ionic liquid functionalized SiO_2_ nanocomposites (SiO_2_-MILSmCl_4_ **a**, SiO_2_-MILZrCl_5_ **b**, SiO_2_-MILCoCl_3_ **c**, SiO_2_-MILEuCl_4_ **d**, SiO_2_-MILFeCl_4_ **e**, and SiO_2_-MILCl **f**) were employed as heterogeneous catalysts (0.15 g for each) for the solvent-free cyclization reaction of CO_2_, propylene oxide and aniline to produce 5-methyl-3-phenyloxazolidin-2-one at 70 °C and 0.1 MPa CO_2_ (Fig. 8a). Different catalysts were tested to demonstrate the effectiveness of supported ionic liquids in the cyclization reaction, the results reflected that SiO_2_-MILZrCl_5_ (yield of 93% and selectivity of 99.4%) and SiO_2_-MILEuCl_4_ (yield of 90% and selectivity of 98.7%) exhibited the excellent performance within only 3 h, outperforming other heterogeneous samples including SiO_2_-MILSmCl_4_ (yield of 83% and selectivity of 98.1%), SiO_2_-MILCoCl_3_ (yield of 46% and selectivity of 87.2%), SiO_2_-MILFeCl_4_ (yield of 65% and selectivity of 91.5%) and SiO_2_-MILCl (yield of 52% and selectivity of 90.1%). As illustrated in Fig. 7a, the catalysis efficiency decreased in the order of SiO_2_-MILCl < SiO_2_-MILCoCl_3_ < SiO_2_-MILFeCl_4_ < SiO_2_-MILSmCl_4_ < SiO_2_-MILEuCl_4_ < SiO_2_-MILZrCl_5_. The differences in activity may be ascribed to the differences in the metal loadings (Table S3) and ionic liquid loadings (Table S4) of the as-synthesized nanocomposites and the synergistic effect between metal-containing anions/cation sites of the functionalized ionic liquid and hydroxyl sites of SiO_2_. As depicted in Tables S3 and S4, the nanocomposites SiO_2_-MILZrCl_5_ and SiO_2_-MILEuCl_4_ demonstrate both high metal loadings (9.07 wt% and 8.95 wt%) and high-density ionic liquid sites (2.21 mmol g^− 1^ and 2.13 mmol g^− 1^), highlighting the catalytic ability of local CO_2_ conversion near active sites under mild condition. The results also indicated that the non-metal anion-functionalized SiO_2_-MILCl exhibited poor catalytic performance, reflecting the necessity of the metal chloride anion ionic liquid functionalized SiO_2_ nanocomposites. In contrast, the catalytic performance of representative bulk ionic liquids (MILZrCl_5_, MILSmCl_4_, and MILEuCl_4_) (0.15 g for each) were also evaluated the catalysis efficiency of the cyclization. Under the same catalytic conditions, all the bulk ionic liquids exhibited significantly lower activity, suggesting that the anion-exchanged functional ionic liquids within the silica framework are highly active for the reaction. Such results further validated the superior catalytic performance of SiO_2_-MILZrCl_5_ under mild condition. To establish the optimal reaction conditions for SiO_2_-MILZrCl_5_, the effect of the reaction parameters including the catalyst amount, reaction temperature and reaction time were examined (Fig. 8b-d). As shown in Fig. 8b, increasing the catalyst amount from 0 to 0.15 g significantly enhanced the catalytic activity. However, no significant improvement was observed with further increase in catalyst amount. Therefore, 0.15 g catalyst is the optimal choice. The effect of reaction temperature was also assessed. The results, presented in Fig. 8c, demonstrated significant variations in yield as the reaction temperature increased from 30 °C to 70 °C. Remarkably, at 70 °C, a high product yield of 93% and an excellent selectivity of 99.4% were achieved. After that, the dependence on the reaction temperature is limited. Similarly, it was found that the yield of product increased with the reaction time increased and reached the maximum value at 3 h, and no significant improvement was observed with further increase in reaction time (Fig. 8d).

**Fig. 9:**
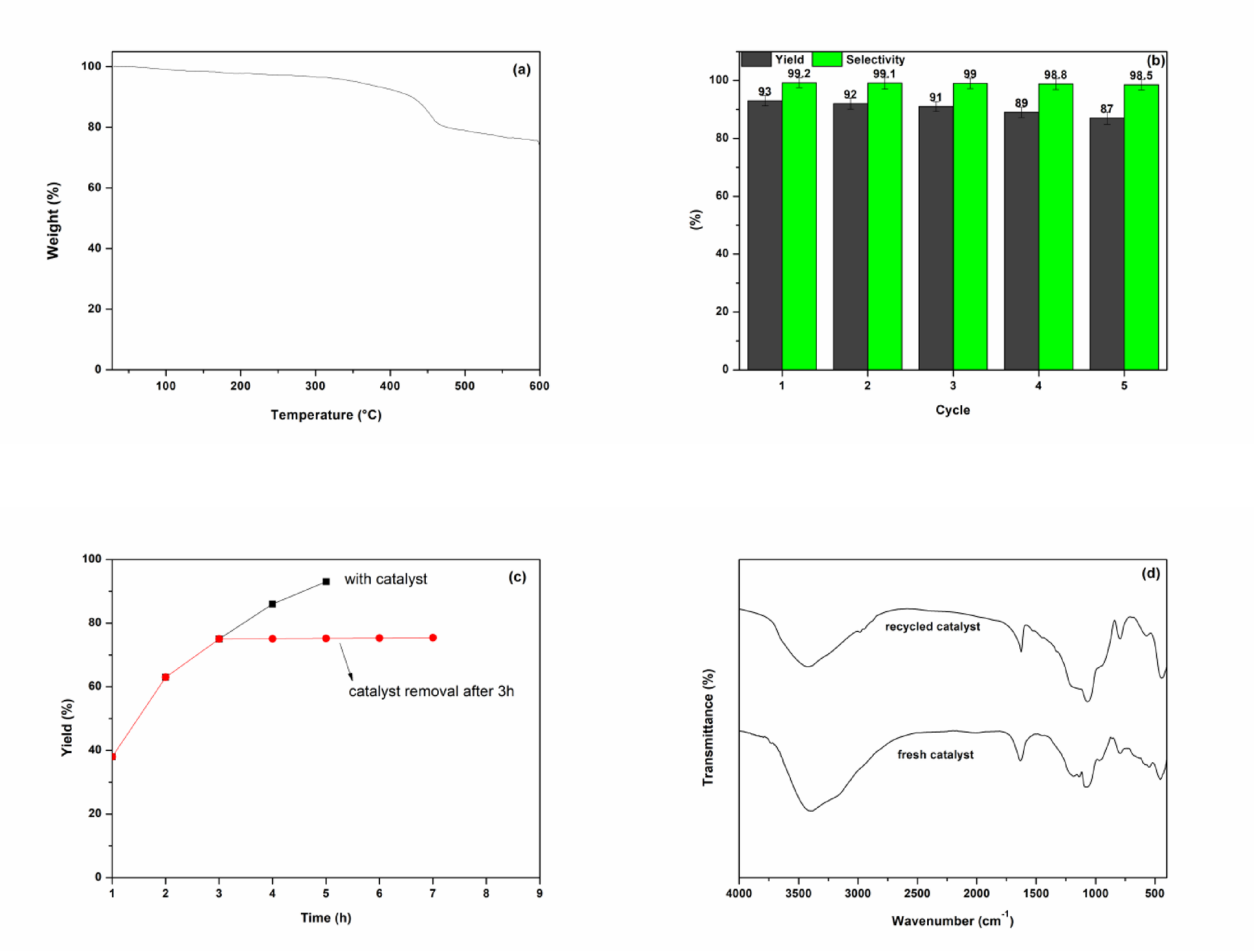
(a) TGA curve of SiO_2_-MILZrCl_5_ catalyst, (b) catalytic activity of the recycled SiO_2_-MILZrCl_5_ catalyst, (c) hot filtration test during the catalytic process, (d) FT-IR spectra of fresh SiO_2_-MILZrCl_5_ and recovered SiO_2_-MILZrCl_5_ after 5th run

Thermogravimetric analysis (TGA) measured the stability of the SiO_2_-MILZrCl_5_ catalyst in the temperature range of 25 ~ 600 °C (Fig. 9a). The initial weight loss of 1.24% observed below 150 °C were ascribed to the desorption of adsorbed water and solvent molecules. The major weight loss of 26.23% occurring above 200 °C were due to the thermal decomposition of the organic ionic liquid segments. TGA confirmed that the catalyst exhibit sufficient thermal stability in the CO_2_ conversion. The recovery and reuse of catalysts are crucial for evaluating the performance of heterogeneous catalyst. The recycling performance of SiO_2_-MILZrCl_5_ was investigated in the model cyclization reaction of CO_2_, propylene oxide and aniline under optimal conditions. As depicted in Fig. 9b, a five-cycle recycling experiment shows that SiO_2_-MILZrCl_5_ retains the catalytic performance with negligible loss, suggestive of its excellent stability. The ICP-AES analysis of the filtrate revealed the almost absence of Zr metal, ruling out catalyst sites leaching (Table S5). Moreover, a hot filtration experiment was performed (Fig. 9c). The reaction was halted after 3 h, and the catalyst was promptly removed via hot filtration. The reaction mixture was then allowed to proceed under the same conditions for an additional 4 h. It can be seen that there are no obvious conversions in the reaction, suggesting the heterogeneous feature of SiO_2_-MILZrCl_5_ catalyst and an absence of catalyst leaching. The FT-IR spectrum (Fig. 9d) and XPS spectroscopy (Fig. S4) of the recovered SiO_2_-MILZrCl_5_ after five runs overlapped with that of fresh one indicating its durability under solvent-free condition. Furthermore, SEM images (Fig. 3g) also revealed no significant changes in morphology, responding to its good stability and recyclability.

**Fig. 10:**
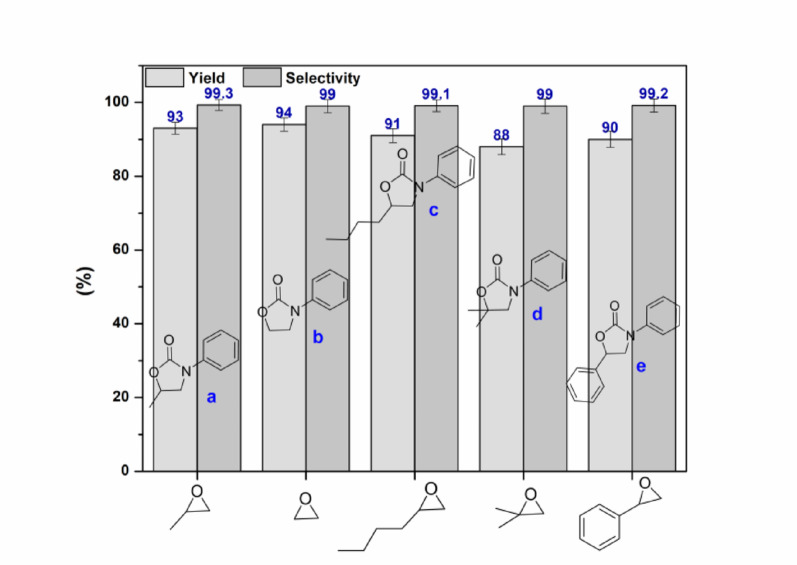
Catalytic fixation of CO_2_ into 2-oxazolidinones (10 mmol epoxide, 4.5 mmol aniline, 0.15 g SiO_2_-MILZrCl_5_, 0.1 MPa CO_2_, 70 °C, 3 h)

Furthermore, a comparison with recent reports on heterogeneous catalytic systems for CO_2_ cyclization with epoxide and aniline is provided in Table S6. Notably, many of the systems require significantly prolonged reaction times and cocatalysts. It can be found that SiO_2_-MILZrCl_5_ is a promising highly active heterogeneous catalyst in the reaction. The good catalytic performance of SiO_2_-MILZrCl_5_ prompts us to examine the substrate compatibility with other epoxides. The cyclization reaction was extended to other epoxides, with aniline and CO_2_ under optimized reaction parameters. As shown in Fig. 10, the SiO_2_-MILZrCl_5_ catalyst can effectively catalyze the coupling of epoxides, aniline and CO_2_ to produce the respective 2-oxazolidinones in high yields (88–94%) and excellent selectivities (≥ 99%), reflecting broad substrate compatibility of SiO_2_-MILZrCl_5_.

**Scheme 3:**
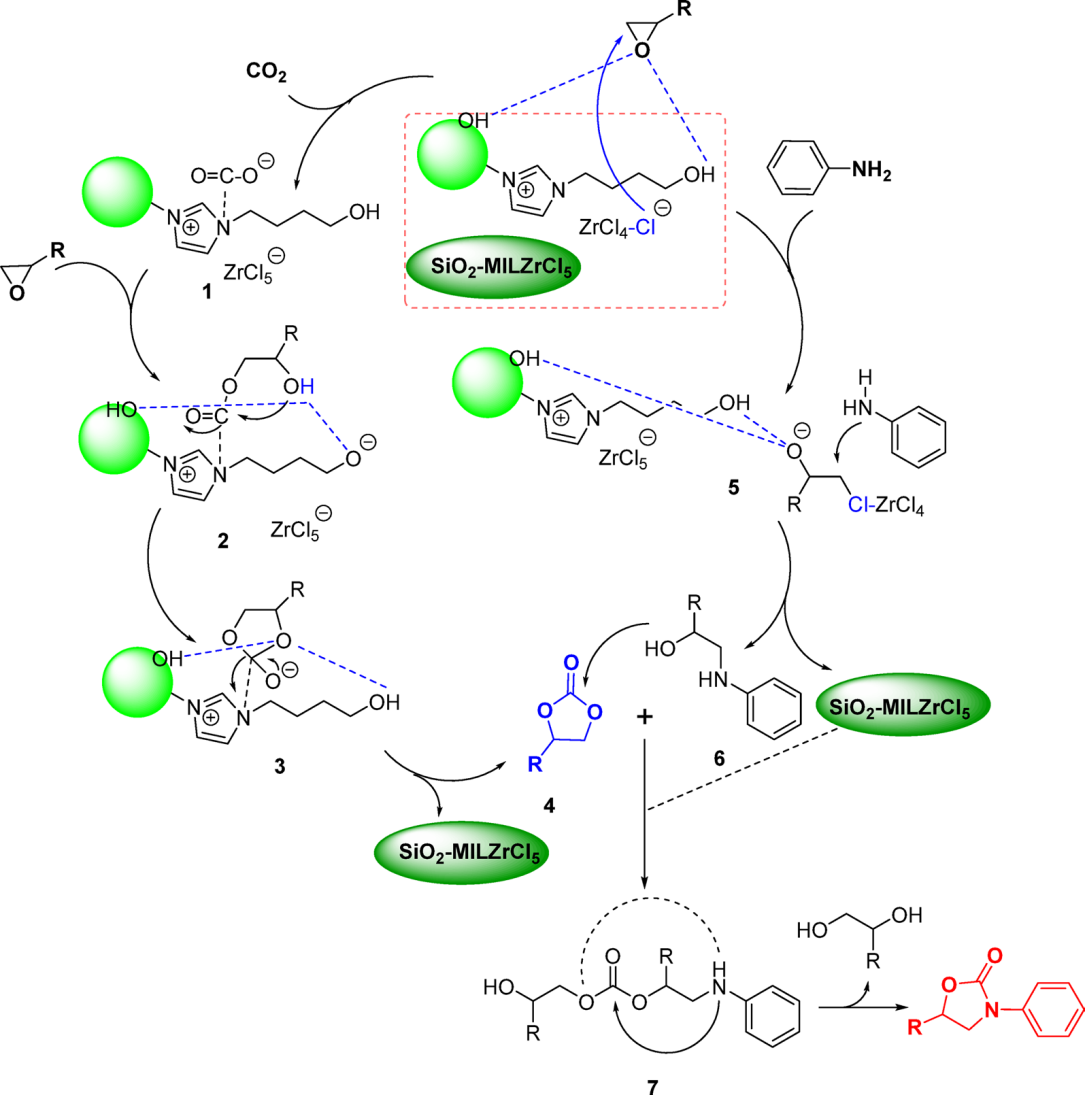
Possible mechanism for the CO_2_ cyclization reaction

Combining with experimental results and previous reports [18–23], a possible reaction mechanism for the CO_2_ cyclization is proposed as illustrated in Scheme 3. CO_2_ is initially captured and activated by the cation of ionic liquid to form carbonate species **1**. The epoxide can be activated through hydrogen bonding of hydroxyl groups, and the activated complex facilitates nucleophilic attack by the carbonate species **1** at its less sterically hindered C atom, resulting in ring opening to form an intermediate **2**, subsequent nucleophilic substitution to form the alkyl carbonate anion **3**. The catalytic cycle concludes through intramolecular cyclization to form cyclic carbonate **4** (confirmed by GC analysis) along with hydroxyl liberation and catalyst regeneration. Meanwhile, the other epoxide can be activated through hydrogen bonding of hydroxyl groups, and the activated complex facilitates nucleophilic attack by the ZrCl_4_-Cl anion at its less sterically hindered C atom, resulting in ring opening to form an intermediate **5**, **5** reacted with aniline via a nucleophilic addition to form intermediate β-amino alcohol **6**. Subsequently, the process concluded with a nucleophilic addition reaction of cyclic carbonate **4** and β-amino alcohol **6** and formed an intermediate **7**, thereby regenerating the catalyst and completing the catalytic cycle. The reaction sequence is completed by a final nucleophilically attack and intramolecular cyclization step, affording the desired product 2-oxazolidinone and the 1,2-diol byproduct.

## Conclusion

In summary, we have successfully developed a type of ionic liquid functionalized SiO_2_ nanocomposites for catalyzing the fixation of CO_2_ into 2-oxazolidinones. Systematic investigation of reaction parameters reveals that SiO_2_-MILZrCl_5_ exhibits the best catalytic performance for the CO_2_ cyclization reaction with various epoxides and aniline under low pressure (even atmospheric pressure) and low temperature, achieving the 88 ~ 94% isolated yields and excellent selectivities (99 ~ 99.4%) within a short reaction time of 3 h. The catalyst can be readily recovered and reused with excellent stability. The green protocol is attractive in terms of its simplicity of procedure, easy separation of the catalyst, environmental compatibility, recycle exploitation and high to excellent isolated yields and selectivities. This general synthetic strategy opens up new possibilities for the design of heterogeneous nanoporous imidazolium-based ionic moieties, endowing broader applications in the catalytic conversion of CO_2_ to valuable chemicals.

## Supplementary Information

Below is the link to the electronic supplementary material.

Supplementary Material 1.

## Data Availability

The data that support the findings of this study are available from the corresponding author upon reasonable request.
