# Supplementary material for: Efficient and sustainable fixation of CO2 into 2-oxazolidinones utilizing ionic liquid functionalized SiO2 nanocomposites
Source: BMC Chem. 2025 Sep 30;19(1):268. doi: 10.1186/s13065-025-01627-7 (PMC12481948; doi:10.1186/s13065-025-01627-7)
Supplement: Supplementary file 1 — Supplementary Material 1. [file 13065_2025_1627_MOESM1_ESM.pdf]

Supplementary Information

Efficient and sustainable fixation of CO_2 into 2-oxazolidinones utilizing ionic liquid functionalized SiO_2 nanocomposites

Yulin Hu[1], Shiyao Lin[1], Xiaobing Liu[2]*

[1]*College of Chemistry and Chemical Engineering, Anshun University, Anshun 561000, China*

[2]*College of Chemistry and Chemical Engineering, Jinggangshan University, Ji'an 343009, China*

*Correspondence**:**

Xiaobing Liu

liuxiaobing805@163.com; ylhanshun@126.com

Experimental

Materials and apparatus

4-(1H-imidazol-1-yl)butan-1-ol was of analytical grade and procured from Achem-Block Chemical Company. Other chemicals were obtained from commercial sources (Sigma-Aldrich) and used without further purification. Fourier transform infrared spectroscopy (FT-IR) analysis was carried out using a Nicolet Nexus 470 spectrometer, with spectra recorded in the range of 4000-400 cm-1 (KBr pellet method). Morphological analysis was conducted using scanning electron microscopy (SEM, Sigma300). Elemental composition and distribution were determined by Energy-dispersive X-ray analysis (EDX) (UltimMax 40) system. X-ray diffraction (XRD) patterns were acquired using a Rigaku Ultima IV diffractometer with Cu Kα radiation. Thermal stability was evaluated by thermogravimetric analysis (TGA) using a METTLER TOLEDO-TGA/DSC-1 instrument under nitrogen atmosphere with a heating rate of 10 °C min^{-1}. The pore structure characteristics were analyzed by N_2 adsorption and desorption curve using a surface area and porosity analyzer (Micromeritics ASAP 2460, USA). X-ray photoelectron spectra (XPS) were obtained using a Thermo Scientific K-Alpha Nexsa instrument with Al Kα radiation. NMR spectra were acquired using a Bruker AVANCE-III 400 MHz spectrometer with $CDCl_3$ as the solvent. Elemental analysis was performed on a Elementar-UNICUBE Analyzer. ICP-AES analysis was carried out using an Optima 2100-DV spectrometer (PerkinElmer), and ICP-OES analysis was carried out using an Avio 200 ICP Optical Emission Spectrometer. The transmission electron microscope (TEM) experiment was carried out on the JEM-2100plus Flash field emission electron microscope (JEOL, Japan), and the acceleration voltage was 200 kV. Chemical yields refer to pure isolated products. The reaction mixture was analyzed by gas chromatography (GC, Agilent 7890A) equipped with an HP-INNO Wax capillary column (60m×0.25 mm×0.5 μm) with a flame ionization detector (FID) to quantify the conversion and selectivity. The conversion of aniline and the selectivity for the product were calculated using the below formula. Conversion (%) = (1- $X_{aniline\ (f)}/X_{aniline\ (i)}$) ×100%, Selectivity (%) = Mass of 2-oxazolidinone/total mass of the product ×100%, where $X_{aniline\ (i)}$ and $X_{aniline\ (f)}$ are the initial and final concentration of aniline, respectively.

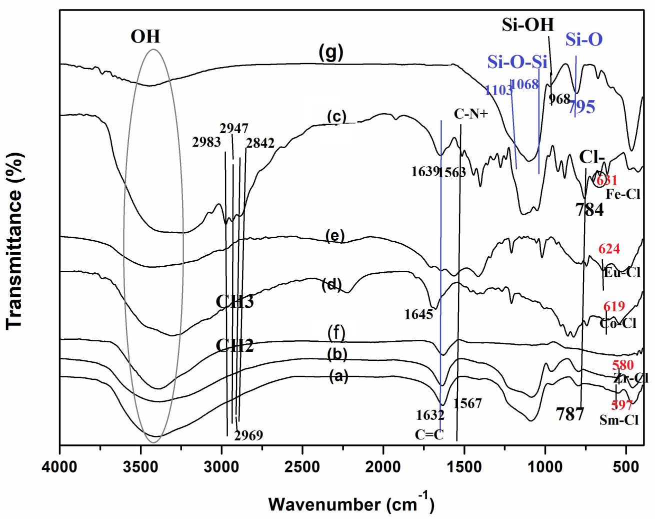

Fig. S1. FT-IR spectra of (a) $MILSmCl_4$, (b) $MILZrCl_5$, (c) $MILCoCl_3$, (d) $MILEuCl_4$, (e) $MILFeCl_4$, (f) MILCl and (g) SiO_2.

SiO_2-$MILSmCl_4$

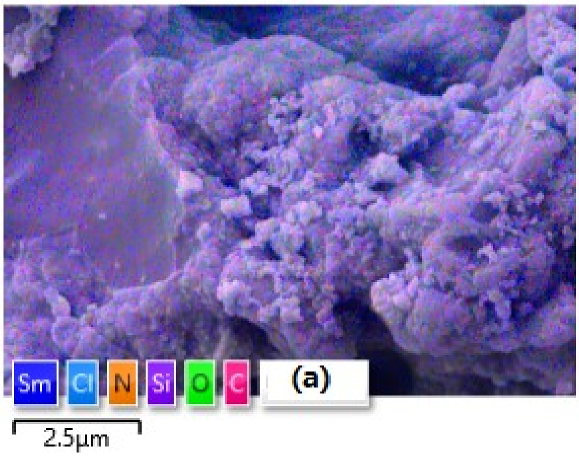

Si Kα1

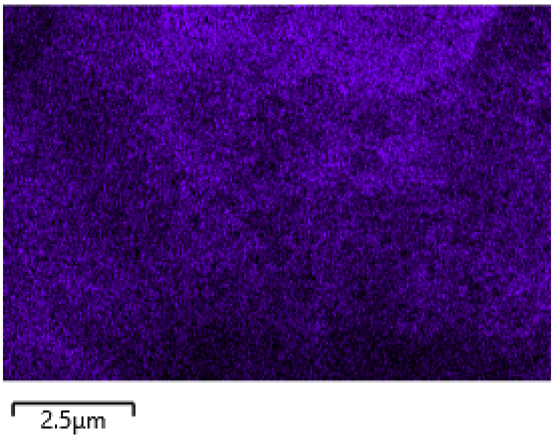

O Kα1

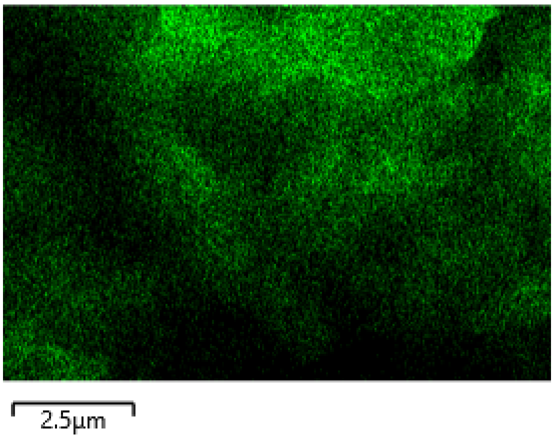

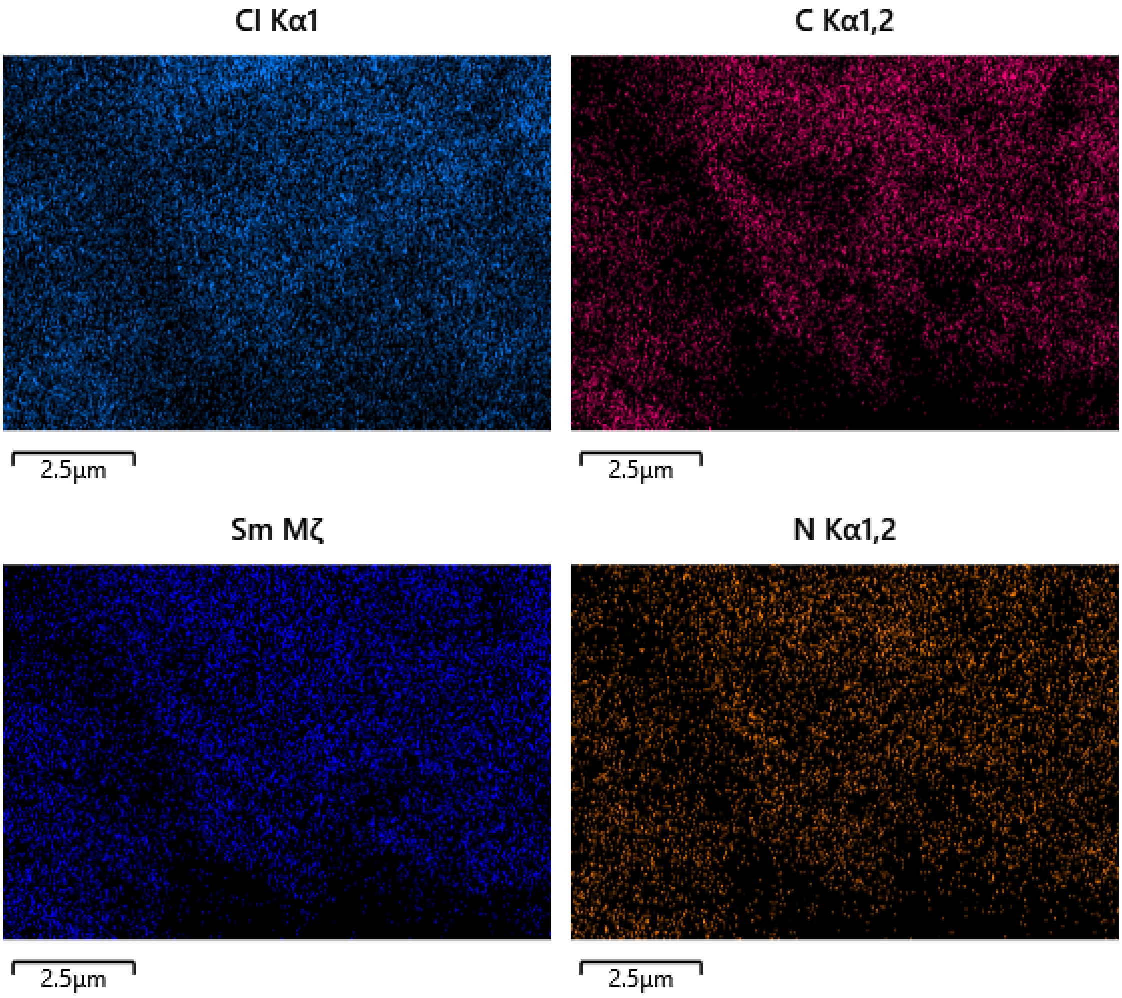

SiO_2-MILZrCl$_5$

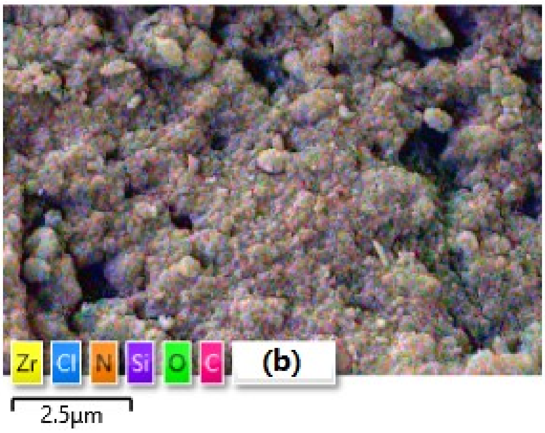

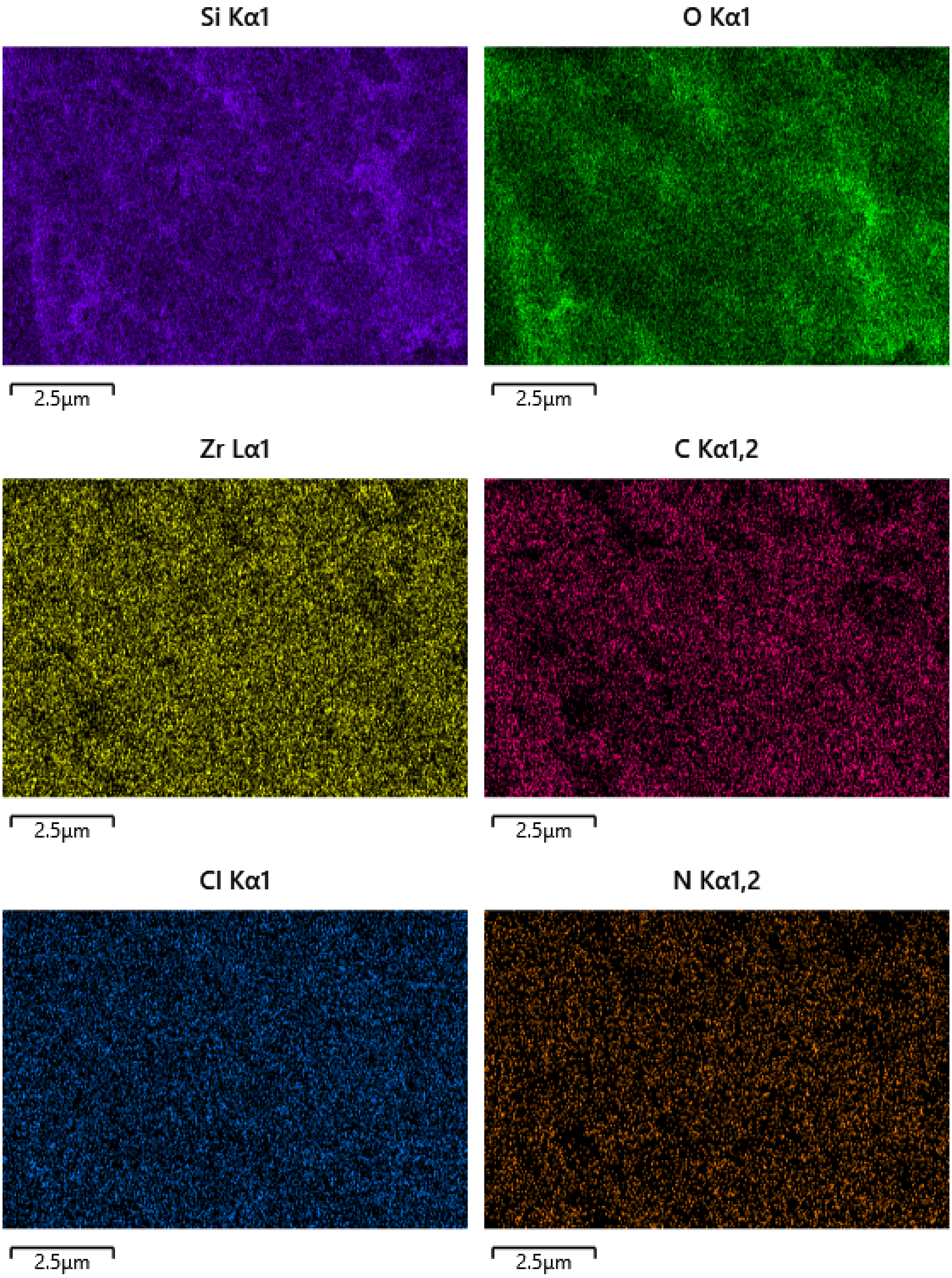

SiO_2-MILCoCl$_3$

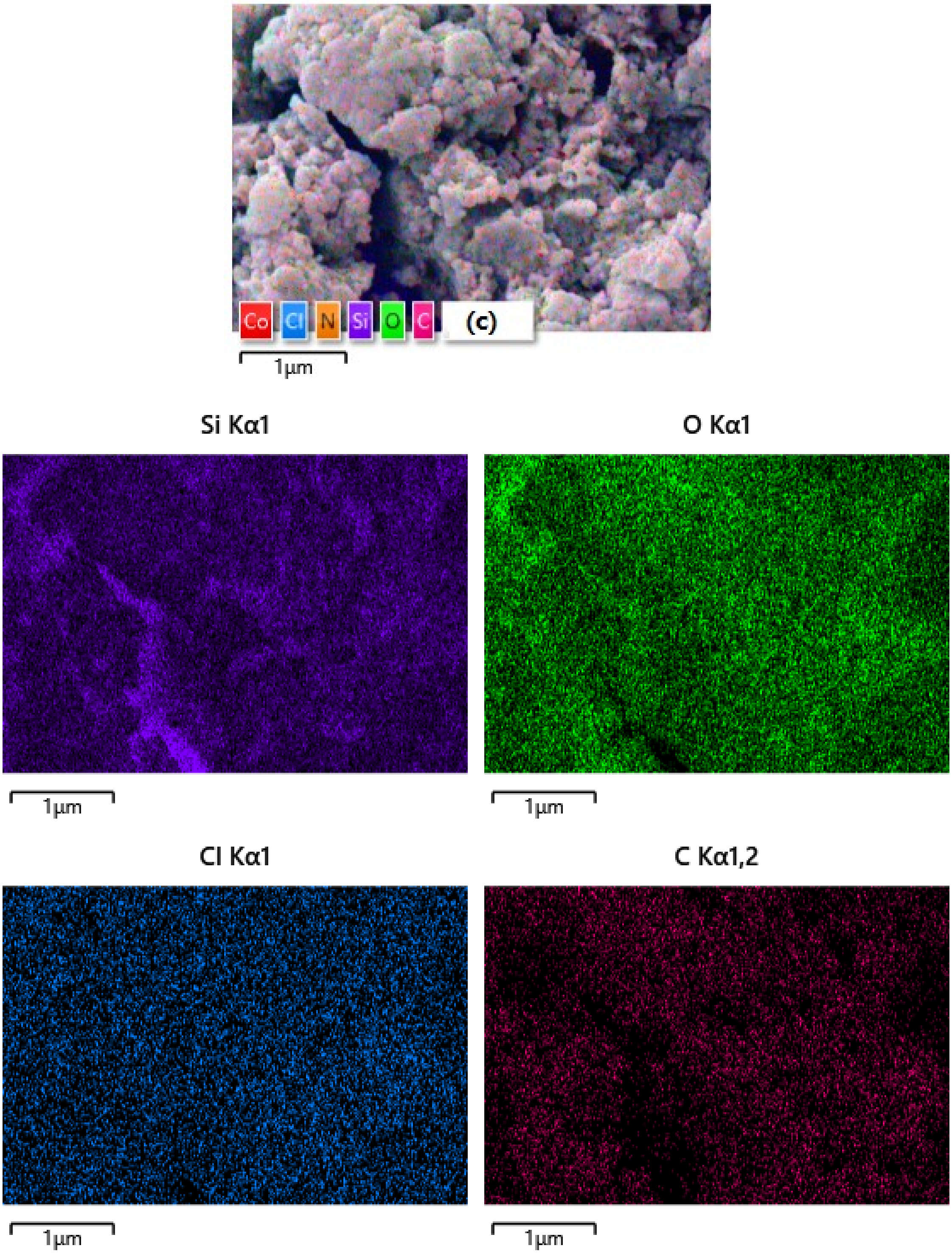
Co
Cl
N
Si
O
C
(c)
1µm
Si Kα1
O Kα1
1µm
1µm
Cl Kα1
C Kα1,2
1µm
1µm

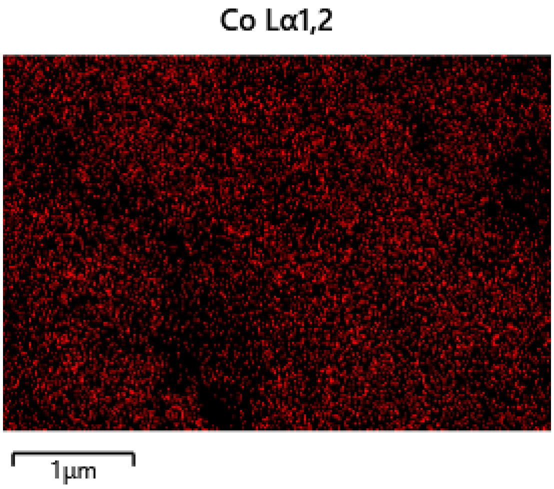

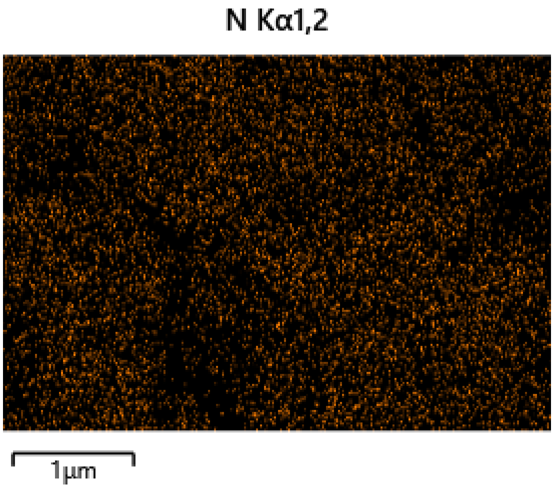

SiO_2-MILEuCl$_4$

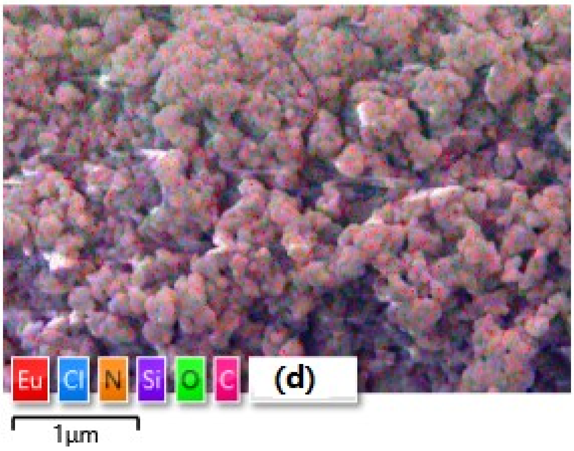

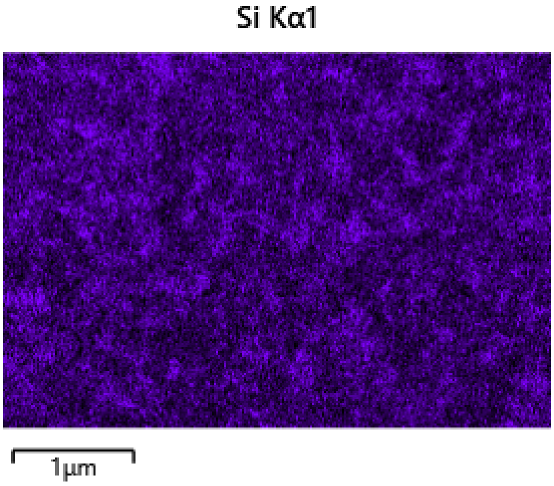

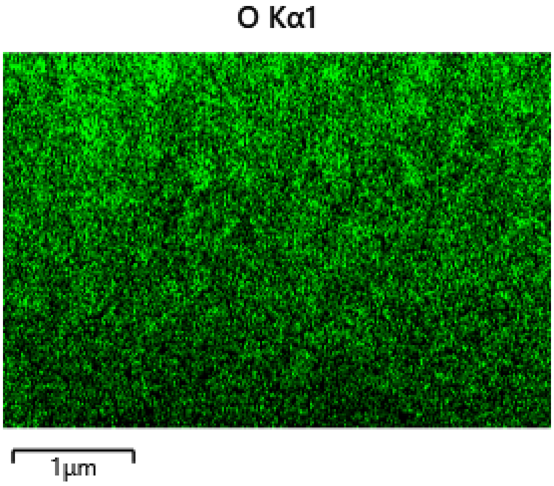

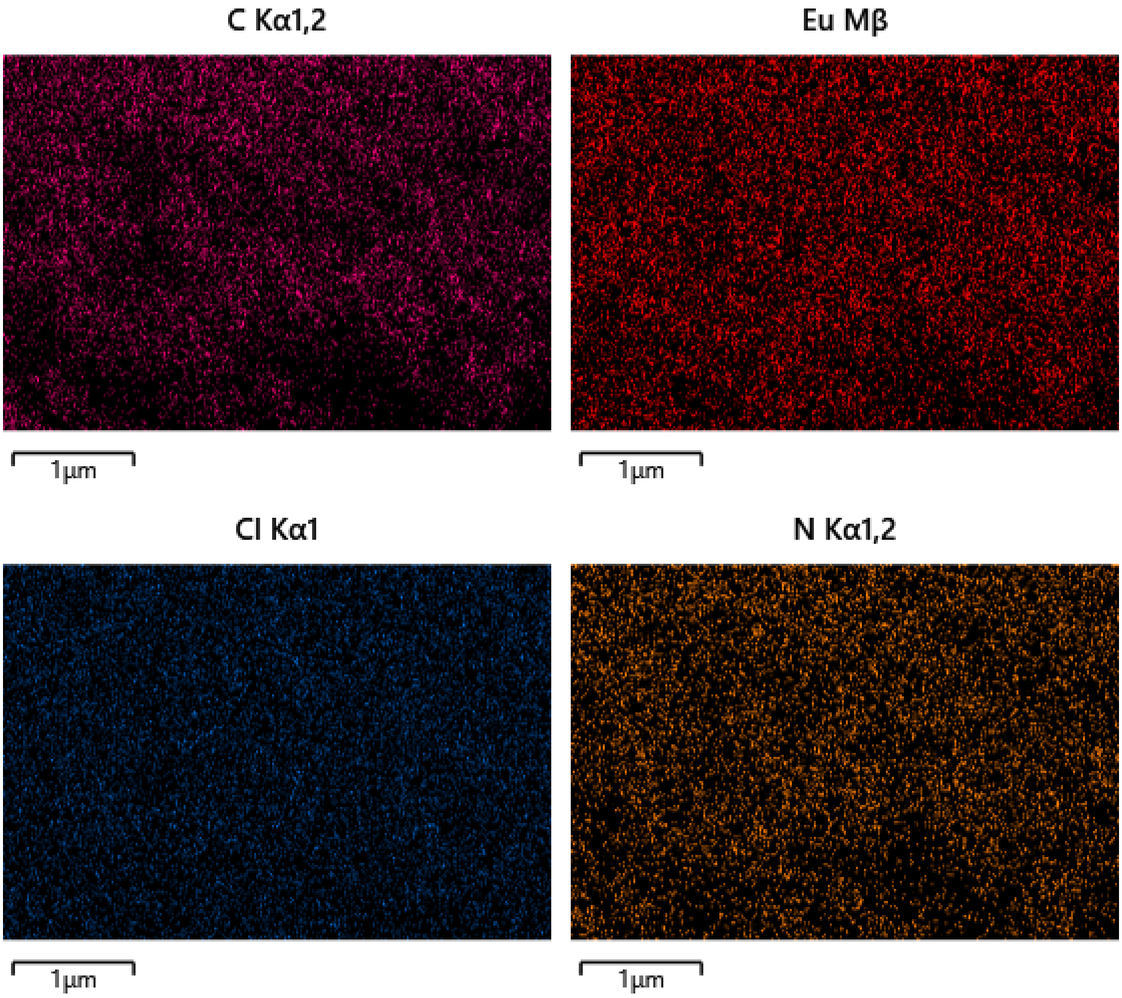

SiO_2-MILFeCl$_4$

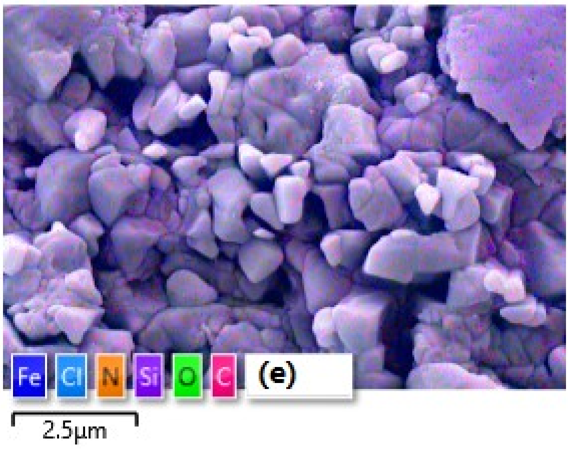

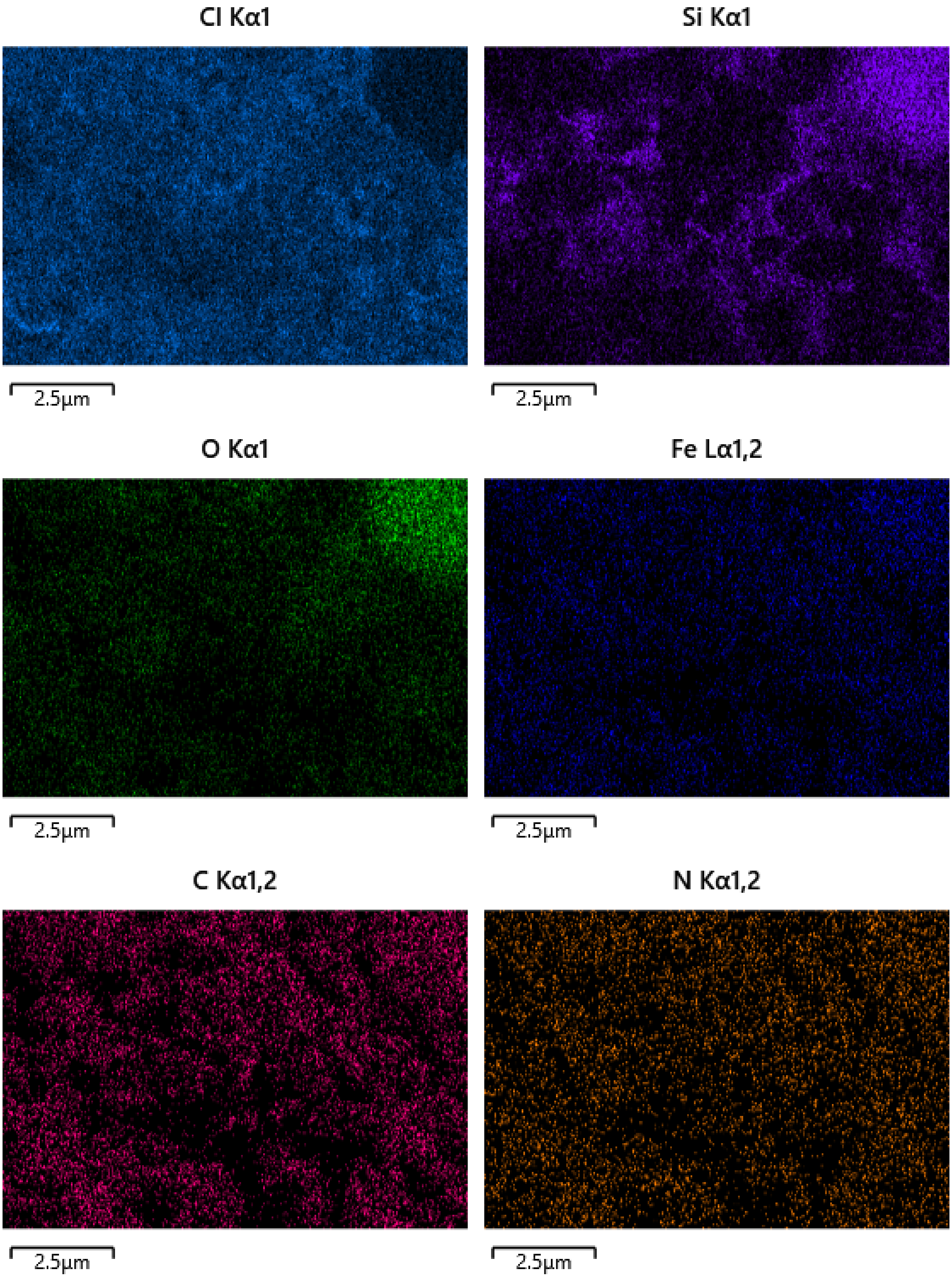
Cl Kα1
Si Kα1
2.5μm
2.5μm
O Kα1
Fe Lα1,2
2.5μm
2.5μm
C Kα1,2
N Kα1,2
2.5μm
2.5μm

SiO_2-MILCl

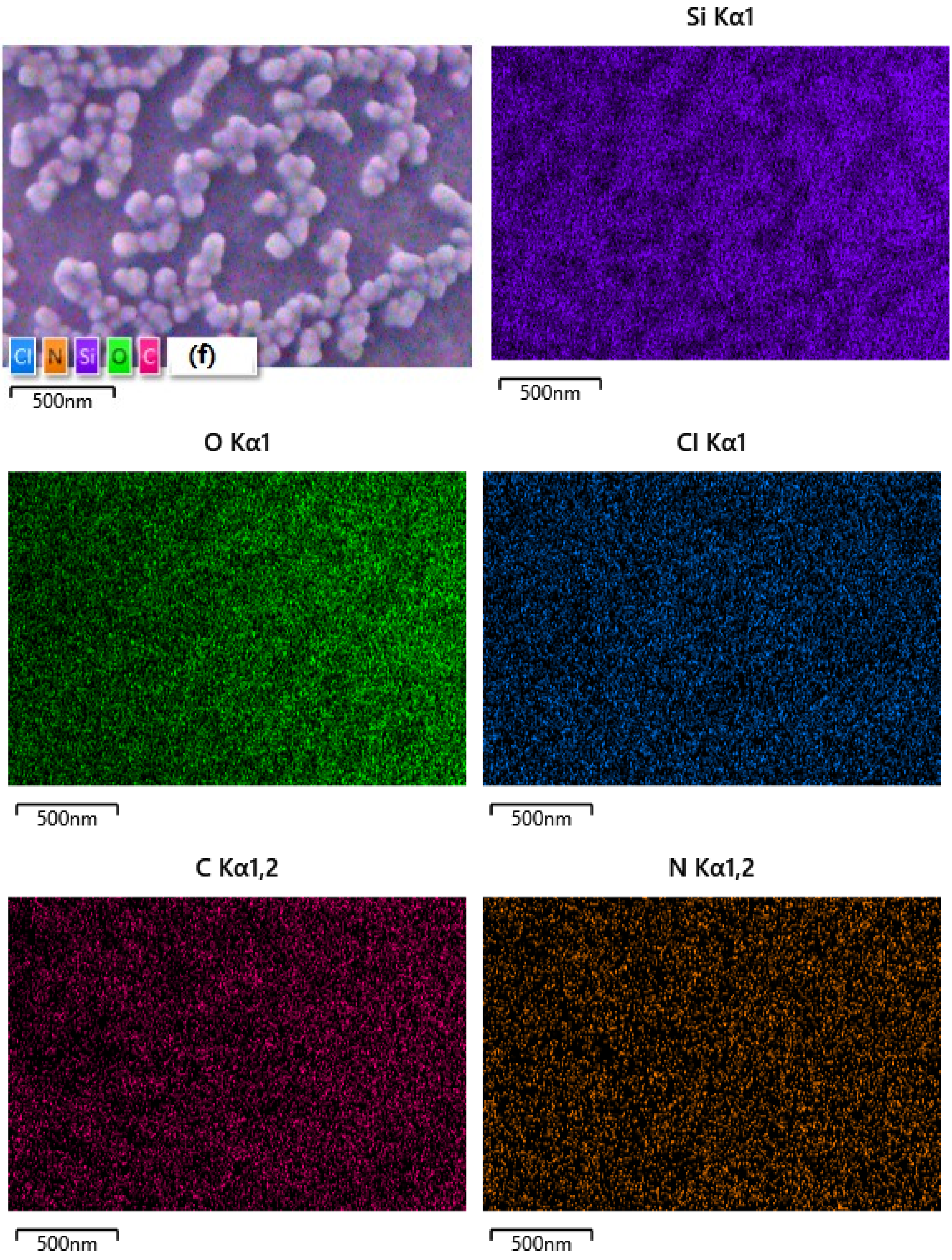

Fig. S2 Elemental mapping images (a) SiO_2-MILSmCl_4, (b) SiO_2-MILZrCl_5, (c) SiO_2-MILCoCl_3, (d) SiO_2-MILEuCl_4, (e) SiO_2-MILFeCl_4, and (f) SiO_2-MILCl.

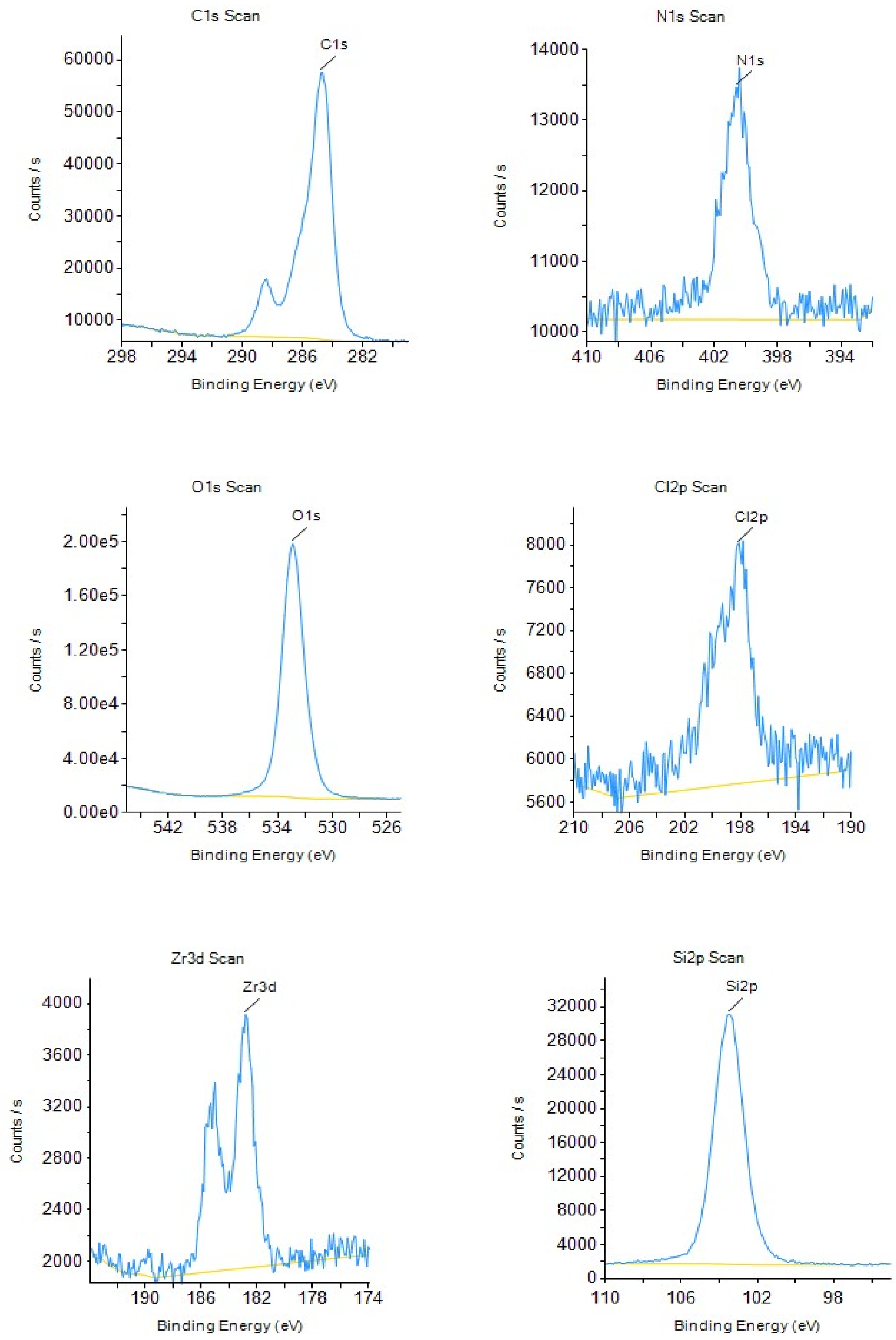

Fig. S3. XPS scan spectra C1s, N1s, O1s, Cl2p, Zr3d, Si2p of SiO_2-MILZrCl_5 catalyst.

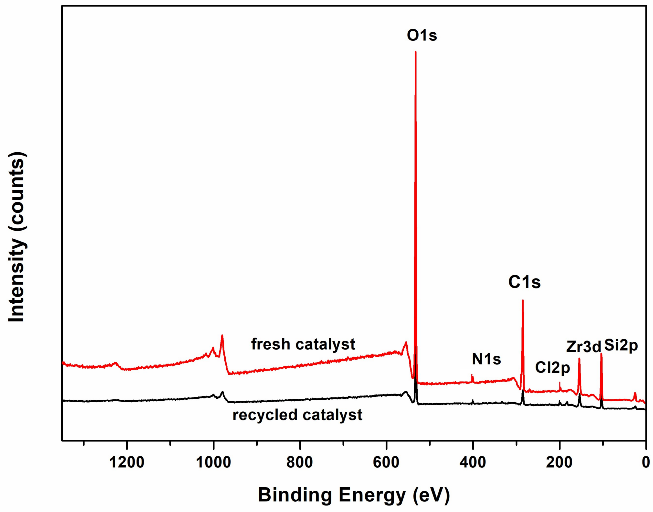

Fig. S4. XPS spectra of fresh and reused IL-SbF_6@nano-SiO_2 catalyst.

Table S1 EDX elemental composition of SiO_2-MILanion nanocomposites

Sample	Si (wt.%)	C (wt.%)	O (wt.%)	M (wt.%)
SiO_2-MILSmCl_4	25.14	16.80	26.53	Sm 9.57
SiO_2-MILZrCl_5	26.43	20.32	25.85	Zr 9.35
SiO_2-MILCoCl_3	25.64	18.50	23.27	Co 8.23
SiO_2-MILEuCl_4	28.41	15.32	25.33	Eu 8.91
SiO_2-MILFeCl_4	28.52	25.64	21.37	Fe 5.24
SiO_2-MILCl	30.69	27.47	29.48	-

Table S2. BET surface area of SiO_2-MILZrCl_5 and SiO_2-MILCl

Sample	A_{BET} (m^2/g)[a]	V_p (cm^3/g)[b]	d_{pBJH} (nm)[c]
SiO_2-MILZrCl_5	182.54	0.26	4.56
SiO_2-MILCl	279.23	0.38	4.17

[a]Specific surface area obtained using a Brunauer-Emmett-Teller (BET) plot. [b]Specific pore volume. [c]Pore diameter obtained using the Barrett-Joyner-Halenda method.

Table S3. Metal loadings of the samples[a]

Sample	Metal loading (wt.%)
SiO_2-MILSmCl_4	Sm (9.13)
SiO_2-MILZrCl_5	Zr (9.07)
SiO_2-MILCoCl_3	Co (6.48)
SiO_2-MILEuCl_4	Eu (8.95)
SiO_2-MILFeCl_4	Fe (5.74)

[a]Determined by ICP-OES.

Table S4. Ionic liquid loading of the samples[a]

Sample	Ionic liquid loading (mmol/g)[a]	Nitrogen loading (wt.%)[b]
SiO_2-MILSmCl_4	1.89	4.15
SiO_2-MILZrCl_5	2.21	4.94
SiO_2-MILCoCl_3	1.75	4.03
SiO_2-MILEuCl_4	2.13	4.73
SiO_2-MILFeCl_4	1.76	4.09
SiO_2-MILCl	3.07	5.12

[a]Calculated loadings of ionic liquids based on nitrogen content as determined by elemental analysis.

[b]Determined by elemental analysis.

Table S5. ICP-AES analysis of metals in the recovered filtrate[a]

Solid catalyst	Recovered filtrate[a]
first recovered SiO_2-MILZrCl_5	Zr (0.00012 wt.%)
third recovered SiO_2-MILZrCl_5	Zr (0.00029 wt.%)
fifth recovered SiO_2-MILZrCl_5	Zr (0.00046 wt.%)

[a]Determined by ICP-AES.

Table S6. Comparison of the performance of CO_2 cyclization over various catalytic systems.

Entry	Catalytic system	Conditions	Yield/Selectivity (%)	Ref.
1	heterometallic [$Ln^{III}{}_2Co^{II}{}_5$] clusters (0.3 mmol)/TBABr (0.5 mmol)	propylene oxide (0.5 mmol), aniline (0.6 mmol), CO_2 (0.1 MPa), DMF (2.0 mL), 80°C, 10 h	92/-	[15]
2	octanuclear Tb(III)-based cluster (0.2 mol%)/TBAI (0.1 mmol)	propylene oxide (6 mmol), aniline (2 mmol), CO_2 (0.1 MPa), 70°C, 10 h	82/-	[17]
3	Ce@PCN-777 (60 mg)	1,2-epoxyhexane (4 mmol), aniline (1 mmol), CO_2 (0.1 MPa), 80°C, 10 h	83/100	[18]
4	PVA-DFNT/Ni NPs (1 mg)	aniline (1.0 mmol), ethylene oxide (10 mmol), CO_2 (1 MPa), 100 °C, 1 h	97/-	[20]
5	K_3PO_4 (43 mg)	styrene oxide (5 mmol), aniline (1 mmol), CO_2 (0.1 MPa), DMF (2 mL), 130°C, 19 h	98/76	[21]
6	UiO-66-40 (70 mg)	propylene oxide (6.0 mmol), aniline (2.0 mmol), CO_2 (1 bar), 85 °C, 12 h	90/-	[22]
7	Organocatalyst (0.05 mmol)/ DBU (0.4 mmol)	aniline (2 mmol), propylene oxide (2 mL), 90 °C, CO_2 (5 bar), 4 h	95/-	[23]
8	Ni-MOF (2.5 mol%)/TBAB (0.020 mmol),	propylene oxide (0.2 mmol), aniline (0.2 mmol), CO_2 (0.1 MPa), 80°C, 12 h	100% conversion	[24]
9	SiO_2-MILZrCl_5 (0.15 g)	propylene oxide (10 mmol), aniline (11 mmol), CO_2 (0.1 MPa), 70 °C, 3 h	93/99.4	This work

NMR spectra of 2-oxazolidinones products

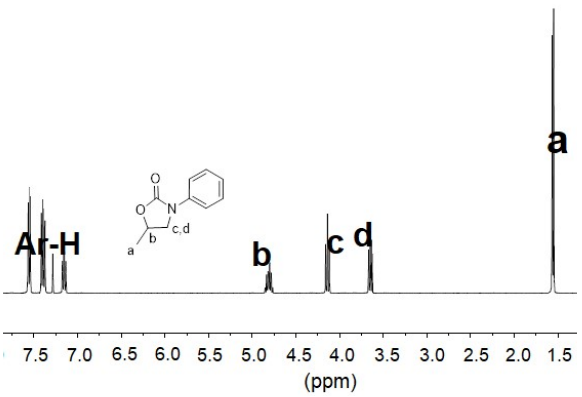

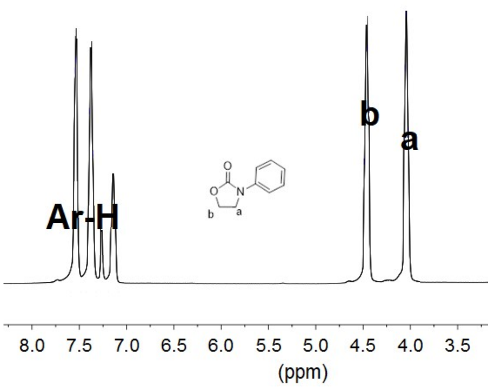

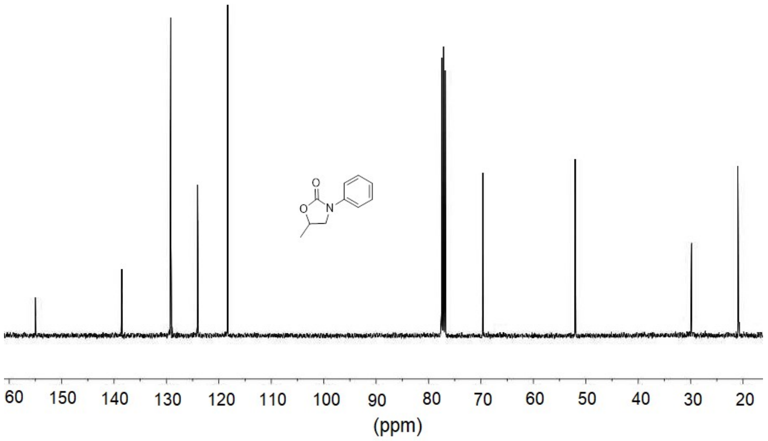

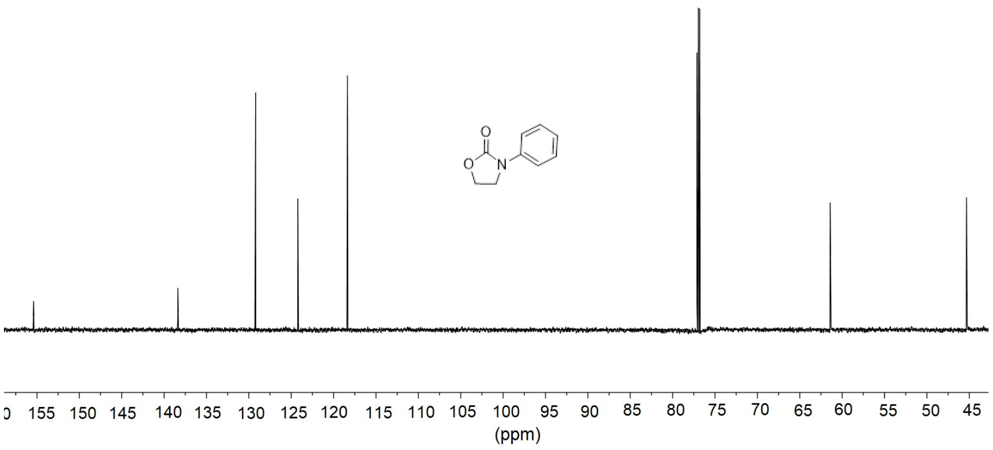

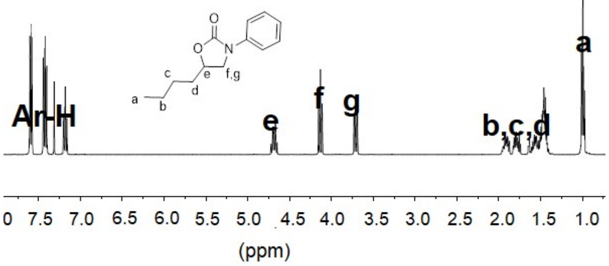
Ar-H
e
f
g
b,c,d
a
a
b
c
d
e
f,g
O
N
(ppm)

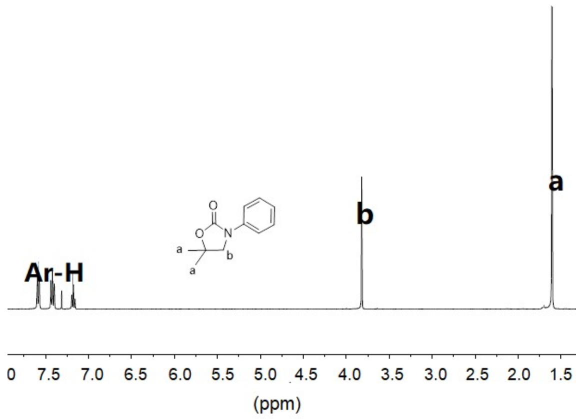
Ar-H
b
a
O
N
(ppm)

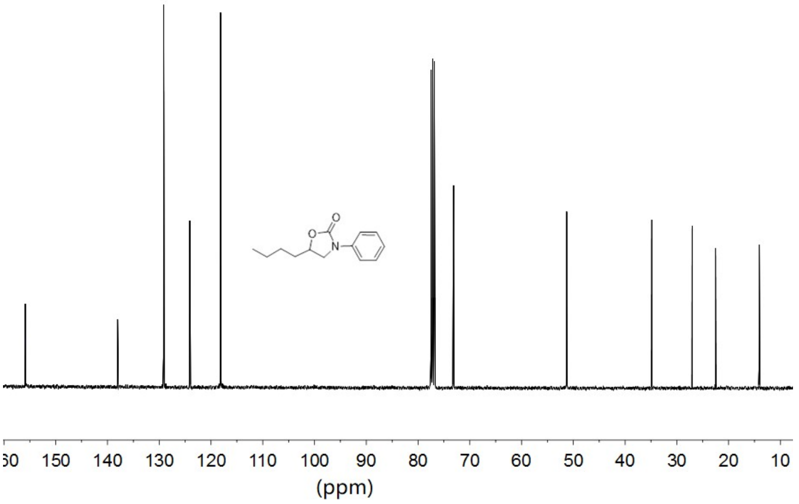
O
N
(ppm)

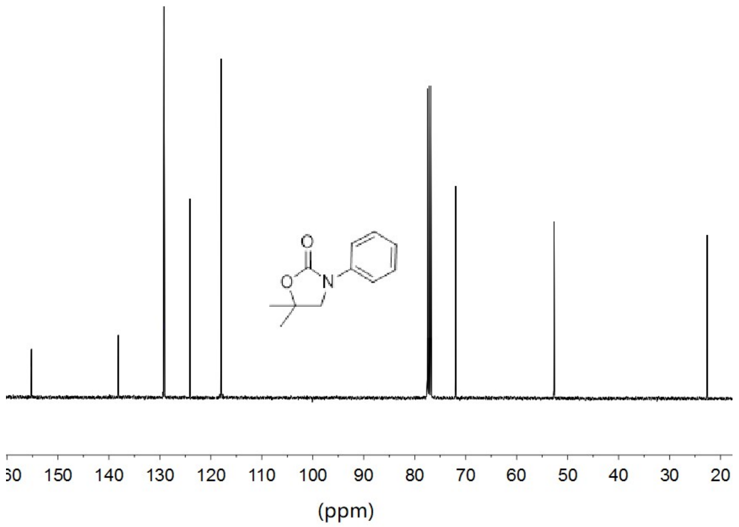
O
N
(ppm)

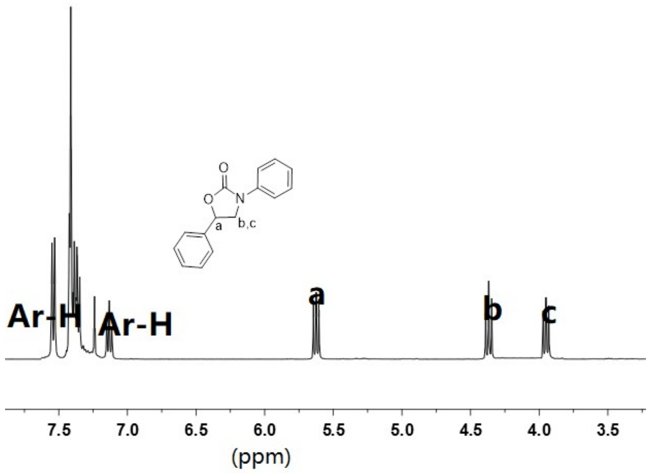
O
O
N
a
b,c
a
b
c
Ar-H
Ar-H
7.5
7.0
6.5
6.0
5.5
5.0
4.5
4.0
3.5
(ppm)

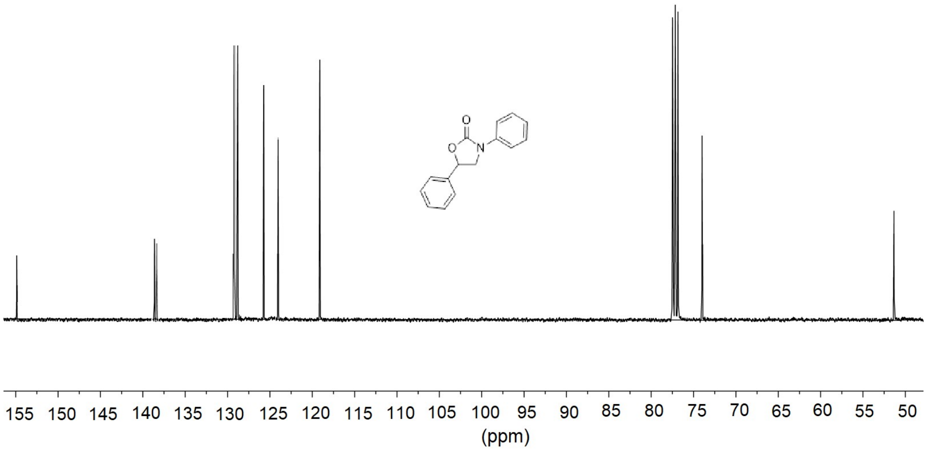
O
O
N
155 150 145 140 135 130 125 120 115 110 105 100 95 90 85 80 75 70 65 60 55 50
(ppm)
